# Unlocking the potential of rice bean (*Vigna umbellata*): Emerging source of bioactives for future foods and food security

**DOI:** 10.1016/j.jfca.2026.109278

**Published:** 2026-06-03

**Authors:** Salman Ahmed, Nabia Shakeel, Khalaf F. Alsharif, Khalid J. Alzahrani, Michael Aschner, Maria Daglia, Mohamed A. Farag, Haroon Khan

**Affiliations:** a Department of Pharmacognosy, Faculty of Pharmacy and Pharmaceutical Sciences, University of Karachi, Karachi 75270, Pakistan; b Department of Pharmacy, Faculty of Chemical and Life Sciences, Abdul Wali Khan University Mardan, Mardan 23200, Pakistan; c Department of Clinical Laboratories Sciences, College of Applied Medical Sciences, Taif University, P.O. Box 11099, Taif 21944, Saudi Arabia; d Department of Molecular Pharmacology, Albert Einstein College of Medicine, Bronx, NY 10461, USA; e Department of Pharmacy, University of Napoli Federico II, Via D. Montesano 49, Naples, NA 80131, Italy; f International Research Center for Food Nutrition and Safety, Jiangsu University, Zhenjiang 212013, China; g Department of Pharmacognosy, Faculty of Pharmacy, Cairo University, Cairo 11562, Egypt; h Department of Pharmacy, Korea University, Sejong 20019, South Korea

**Keywords:** Rice bean, Climate-smart agriculture, Multi-omics integration, Phytochemical diversity, Nutraceutical and pharmaceutical potential, Food security and sustainable nutrition

## Abstract

Underutilized legumes represent an untapped frontier in food, nutrition, and health security. Among them, rice bean (*Vigna umbellata*) stands out for its nutritional richness, ecological resilience, and therapeutic potential, yet remains marginalized in global food systems. Despite its adaptability to heat, drought, and marginal soils, it suffers from limited phytochemical profiling. Similarly, sparse genomic and omics resources, and weak translational evidence linking bioactives to human health. This review compiles advances in phytochemical mapping, highlighting regional variations in flavonoids, phenolic acids, bioactive peptides, and low-glycemic starch. Comparative positioning against major legumes underscores its potential compositional advantages associated with antioxidant and metabolic relevance. Emerging genomic, transcriptomic, metabolomic, and proteomic studies reveal untapped breeding potential, while systems biology and AI-driven omics integration promise accelerated trait improvement. We argue for positioning rice bean as a climate-smart crop within circular and sustainable farming systems, with roles in nitrogen fixation, metabolic health, and functional food innovation. Bridging policy frameworks, market incentives, and interdisciplinary collaborations is critical to elevate rice bean from orphan status to a strategic crop for nutrition-sensitive agriculture. This narrative review synthesizes current compositional, multi-omics, and systems-level evidence on rice bean (*Vigna umbellata*), without employing a systematic literature search or meta-analytical framework. It is concluded with a translational roadmap, outlining integrative research priorities across plant sciences, pharmacology, and socioeconomics to unlock rice bean’s potential as a cornerstone of future-ready food systems.

## 1. Introduction

Underutilized legumes, often termed “forgotten crops,” are gaining renewed attention as critical contributors to global food security, nutrition, and climate resilience. Despite their centuries-long cultivation, these species remain marginalized in mainstream agriculture and diets, particularly in regions facing persistent protein and micronutrient deficiencies (Kaur et al., 2025). Nutritionally, crops such as Bambara groundnut (*Vigna subterranea*) and rice bean (*Vigna umbellata*) provide high-quality proteins, complex carbohydrates, fibers, vitamins, and minerals, alongside bioactive compounds including flavonoids such as rutin and myricetin that confer antioxidant, anti-inflammatory, and metabolic health benefits (Knez et al., 2023). Agronomically, they thrive under marginal conditions of drought, low-fertility soils, and temperature extremes. It’s nitrogen-fixing ability enhances soil fertility and reduces dependence on synthetic fertilizers, thereby supporting sustainable intensification of agroecosystems (Ayilara et al., 2022). Beyond their role in agricultural production, these orphan legumes enhance biodiversity, provide vital ecosystem services such as carbon sequestration, and underpin closed-loop and regenerative farming systems. Advocating their cultivation can enhance local rural economies and food sovereignty as they exist in the culinary cultures of many countries in Sub-Saharan Africa and South Asia. In dryland farming systems, the post-harvest challenges, weak value chains, and low agricultural investments serve as barriers to adoption (Vilakazi et al., 2025). From a production and utilization perspective, rice bean remains regionally significant yet statistically underrepresented in global pulse datasets. In Nepal, rice bean is cultivated predominantly in mid- to high-hill agroecosystems (1000–2000 m), where it contributes meaningfully to household protein intake and soil fertility in mixed cropping systems. National agricultural surveys indicate that underutilized legumes, including rice bean, collectively occupy < 5% of total pulse area, despite their critical role in subsistence farming and dietary resilience (Kaur et al., 2025). Similarly, in Northeast India (states such as Nagaland, Manipur, Meghalaya, and Arunachal Pradesh), rice bean is traditionally grown on jhum (shifting cultivation) lands and terrace systems, often intercropped with maize and millets (Ahmed and Jamil, 2024). Although precise crop-wise statistics are fragmented, regional agronomic assessments suggest that rice bean contributes disproportionately to legume diversity relative to its cultivated area, particularly in marginal hill systems (Mishra et al., 2024). In Southern China and parts of Southeast Asia, rice bean cultivation persists largely outside formal markets, being utilized locally as food, fodder, and soil-enriching biomass rather than as a commercial pulse. This disconnects between regional importance and formal production statistics underscores why rice bean remains invisible in policy-driven food system models despite its demonstrated agronomic and nutritional value (Kimani et al., 2025).

Originating from Africa, *V. umbellata*, commonly known as the Italian spear bean, is increasing in demand in India, Myanmar, and some Southeast Asian countries. This is particularly due to the bean’s self-sustaining farming characteristics, especially in poorer regions where the soils lack vital nutrients (Mmbando, 2025). In terms of nutrition, rice beans stand out due to their 16–19 % protein content, which includes balanced essential amino acids like methionine and tryptophan as well as polyunsaturated fatty acids. Bioactive compounds with anti-inflammatory, antidiabetic, and antioxidant qualities, such as quercetin catechins, and phenolic acids, improve these (Ahmed and Jamil, 2024). Because they are an essential part of indigenous communities’ traditional diets and rituals, rice beans continue to exhibit significant cultural value in addition to their health benefits. However, because of poor processing infrastructure, low market visibility, and little investment in breeding, it is still underutilized despite its potential (Mishra et al., 2024).

Genome-scale resources for *V. umbellata*) have only recently emerged, yet they remain sparse compared with well-studied legumes such as mung bean (*V. radiata*) and adzuki bean (*V. angularis*). A land-mark advance was achieved in 2022 with the release of a draft genome assembly (~414 Mb; ~31,276 protein-coding genes), which identified candidate genes associated with flowering, abiotic stress tolerance, and palatability traits (Kaul et al., 2022). Complementary efforts using a high-quality reference genome, coupled with genotype-by-sequencing of 440 landraces, enabled genome-wide association studies (GWAS) to map loci governing flowering time, yield, and adaptation-related genes such as FT, FUL, and PRR3 (Guan et al., 2022).

Beyond its nutritional attributes, rice bean plays a tangible functional role in traditional agroecosystems. In Southern China, it is widely incorporated as a green manure crop, where its rapid biomass accumulation and efficient symbiotic nitrogen fixation enhance soil organic matter and reduce dependence on synthetic fertilizers in low-input farming systems. In the Eastern Himalayan region, particularly across Nepal and Northeast India, rice bean is frequently intercropped with maize on sloping lands (Hlaing et al., 2024). This maize–rice bean association reduces surface runoff, improves soil aggregation, and mit igates erosion during monsoon seasons, while simultaneously providing a secondary protein-rich harvest. Such field-level practices illustrate that rice bean is not merely a fallback subsistence crop, but an active ecological engineer within fragile mountain agroecosystems, an aspect often overlooked in yield-centric evaluations of crop value (Singh et al., 2025). Despite the abundance of bioactive compounds found in rice bean, mechanistic research on the topic is still lacking. Its seeds contain peptides, flavonoids, and fibers that may have anti-inflammatory, antioxidant, and metabolic-regulating qualities, yet not many comprehensive studies have been reported on their health effects. Among the significant gaps are the effects of flavonoids on oxidative stress, the alteration of gut microbiome composition and metabolite fluxes by fibers, and the modulation of inflammatory signaling pathways by peptides derived from rice beans. Additionally, there are few opportunities for equitable agricultural innovation because indigenous knowledge systems and gender perspectives, which women farmers use to manage the cultivation, processing, and culinary traditions of their crops, rarely find their way into developmental frameworks. Repositioning rice bean legume as a key component of climate-resilient and health-promoting food systems may be possible by extending genome-enabled breeding pipelines that clarify its mechanistic nutraceutical potential and integrating it into inclusive CSA frameworks. Reintegrating it into science policy and practice is now required, not optional.

The aim of this narrative review is to integrate current knowledge on the compositional diversity, phytochemical profiles, and emerging omics resources of rice bean (*V. umbellata*), with particular emphasis on its relevance to nutrition-sensitive agriculture and functional food innovation. The scope of the review encompasses phytochemical composition, genomics, transcriptomics, metabolomics, proteomics, and AI-enabled crop design. As a narrative review, this work does not apply systematic search criteria, quantitative synthesis, or formal risk of bias assessment. Furthermore, many health-related inferences discussed herein are derived from compositional, in vitro, animal, or in silico studies, as human clinical evidence for rice bean bioactives remains limited. These constraints highlight the need for future translational and population-based research.

## 2. Taxonomy, evolutionary origins, and underutilized status

### 2.1. Botanical description and classification

**Fig. 1.**
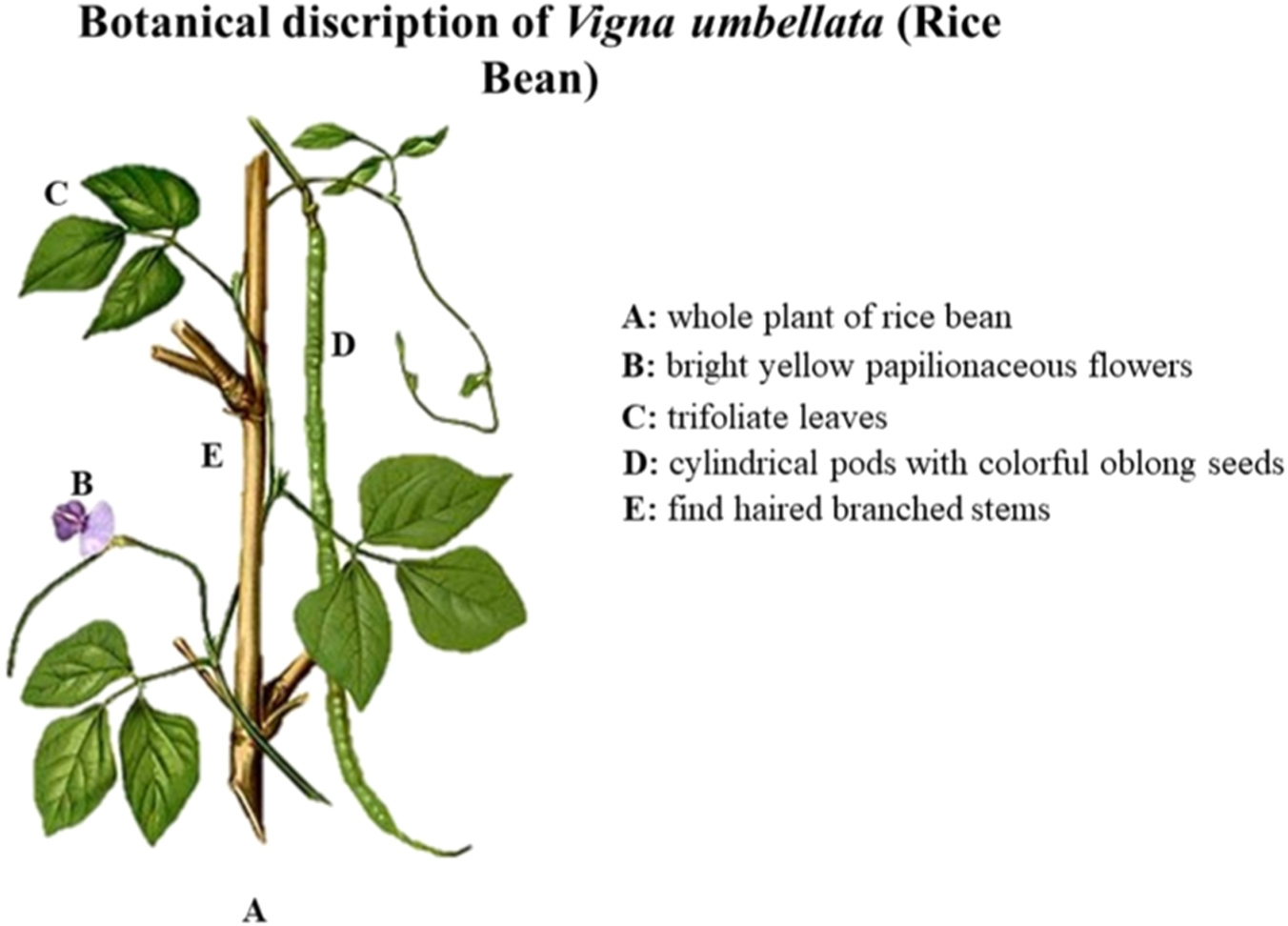
Figure describing botanical description of rice bean plant indicating specific parts my marking (plant diagram taken from web and modified by author).

*Vigna umbellata* (Thunb.) Ohwi & H. Ohashi (Fig. 1), an underappreciated legume of the Fabaceae family, also referred to as rice beans, is prized for its adaptability, resilience, and nutritional potential. Physically, it is an annual twining vine that grows without support in a prostrate to semi-erect manner. Depending on the genotype and environment, its height can range from 30 cm to more than 1.5 m (Isemura et al., 2010). Stems are slender, angular, and pubescent with soft trichomes, green to purple in pigmented varieties, with axillary branching from basal nodes (Wadikar and Bepary, 2020). Leaves are alternate, trifoliate, and ovate–lanceolate (3–12 × 2–7 cm), pubescent on both surfaces; the terminal leaflet is typically larger and more symmetrical than the lateral ones. Petioles, extending up to 12 cm, bear pulvini conferring nyctinastic movements, with lanceolate stipules at the base (Arora et al., 1980). Fruits are cylindrical pods (7.5–12.5 cm), either pubescent or glabrous, dehiscing longitudinally to release 6–10 oblong seeds. Seeds (6–8 × 2–4 mm) display polymorphic colors—red, black, brown, cream, maroon, mottled, or yellow, with a glossy testa, distinct concave hilum, and fleshy cotyledons (Lakshmana et al., 2010). Root systems are deep taproots with extensive laterals and nodulation, forming effective symbioses with *Rhizobium leguminosarum*, thereby contributing substantially to nitrogen fixation and soil fertility, reinforcing their ecological and agronomic value (Wadikar and Bepary, 2020) (Fig. 1).

### 2.2. Global relevance and constraints

#### 2.2.1. Historical neglect and orphan crop status

*V. umbellata* typifies the paradox of orphan legumes: a crop endowed with remarkable nutritional, ecological, and genetic attributes, yet marginalized by historical neglect, fragile value chains, and limited scientific investment. The rice bean was domesticated in Indo-China and spread throughout South and Southeast Asia. It has historically been grown in the marginal hilly and tribal regions of India, Thailand, Vietnam, Laos, and Nepal. Its worldwide reach is still quite small, mainly due to informal seed networks and small-scale subsistence farming systems, even though it can adapt to a variety of agroecologies (Kaul et al., 2023). Its inherent agronomic limitations, such as unpredictable growth patterns, significant variation in seed characteristics, and the presence of antinutritional factors, have also hindered its acceptance, commercialization, and integration into international food and nutritional security plans (Joshi et al., 2007).

#### 2.2.2. Ecological adaptability and climate-smart relevance

Despite its marginalization, rice beans demonstrate exceptional ecological resilience, growing in rainfed, acidic, and drought-prone soils that are typically unsuitable for staple crops. Its adaptability to low-input agroecosystems, particularly in regions dealing with water scarcity and soil degradation, highlights its value as a climate-smart legume (Odeku et al., 2024). Rice beans are not only nutrient-dense, but they also promote sustainability by repairing soil fertility and fixing nitrogen in a symbiotic manner. Their dual use as food and fodder strengthens mixed farming systems. However, in spite of these advantages, major pulses and cereals are prioritized in policy frameworks and extension services, excluding underutilized legumes from the mainstream agricultural agenda.

#### 2.2.3. Nutritional richness vs. commercialization barriers

From a nutritional perspective, rice bean (*V. umbellata*) is an extremely promising but underutilized legume because it combines a high content of macronutrients with bioactive compounds that have useful applications. Vital amino acids like tryptophan and methionine, which are frequently absent from many staple legumes, are added to seeds from a range of genotypes in the Himalayas and Southeast Asia, where protein concentrations can vary from 6% to 25% (Katoch, 2013). Such amino acid balance strengthens its role as an additional protein source when added to cereal-based diets. In addition to proteins, rice beans provide a variety of vitamins and minerals, complex carbohydrates with a low glycemic index, and polyunsaturated fatty acids, such as linoleic and linolenic acids. Rice beans are a promising option for the creation of nutraceuticals and functional foods because of these compounds. These nutritional benefits back the global push to diversify diets and encourage plant-based proteins as part of sustainable diets (Joshi et al., 2007).

Over the past 10 years, there has been a steady increase in scientific interest in rice beans, which is encouraging because molecular and genomic advancements have opened up new avenues for targeted crop improvement (Kaur et al., 2025; Vilakazi et al., 2025). The development of molecular markers, transcriptomic analyses, and draft genome assemblies has enabled breeders to expedite selection for stress tolerance, nutritional quality, and yield. Early-stage breeding programs have been successfully demonstrated to improve seed palatability and consumer appeal, while simultaneously reducing antinutrients such as phytic acid and tannins (Zayed et al., 2025).

Rice beans best illustrate the paradox of an underutilized legume with unrealized global significance. With its nutritional density, ecological adaptability, and genetic resilience, it is a strategic resource for addressing the interrelated issues of micronutrient deficiencies, protein-energy malnutrition, and agricultural sustainability in the face of climate change. However, to fully realize this potential, a coordinated shift in research investment policy priorities and value chain development is required to incorporate rice beans into mainstream agriculture and global food security agendas.

#### 2.2.4. Genetic resources and future opportunities

The *V. umbellata*, a historically underutilized but strategically significant crop with enormous potential for climate-smart agriculture and diversification of the global food supply, sits at the intersection of resilience, sustainability, and nutrition. Only by putting into practice integrated strategies that combine genetic resource conservation, improved agronomy market development, targeted breeding, and enabling policy frameworks can it advance from localized reliance to global agricultural relevance (Gayacharan et al., 2024a). Advances in molecular markers, transcriptomics, comparative genomics, and other genomic and genetic tools offer unprecedented opportunities to expedite trait discovery and enable precision breeding for stress tolerance and yield quality (Mal, 2022). Despite its resilience to drought, pests, and marginal soils, rice bean remains marginalized within the global research agenda, overshadowed by mainstream legumes such as soybean, chickpea, and common bean (Dhillon and Tanwar, 2018). Constraints such as fragmented germplasm collections, indeterminate growth, variable seed colour, and antinutritional factors continue to hinder wider adoption, compounded by limited international investment and collaboration in breeding programs (Zargar et al., 2021). Nonetheless, its dense nutritional profile, being rich in protein, micronutrients, and health-promoting lipids, coupled with environmental adaptability, particularly on degraded lands, positions rice bean as a climate-resilient legume with unique potential to strengthen smallholder livelihoods, food sovereignty, and global food security (Odeku et al., 2024; Katoch, 2013; Dwivedi et al., 2023).

## 3. Climate-smart agriculture (CSA) crop

### 3.1. Climate resilience traits

Because it thrives in a range of challenging agroclimatic conditions, the ecologically adaptable *V. umbellata* holds promise as a crucial element of climate-smart agriculture. Rice beans provide strong protection against biotic threats in addition to their capacity to tolerate abiotic stress. They are naturally resistant to important storage pests like bruchid beetles and field pathogens like leaf spot and powdery mildew, which reduces the need for pesticides and increases post-harvest stability (Pattanayak et al., 2018). Its robust symbiotic nitrogen fixation and efficient nutrient uptake, which boost soil fertility and encourage sustainable rotations, further support its capacity to flourish in degraded acidic and nutrient-deficient soils (Katoch, 2020). The availability of short-duration cultivars that mature in 60–75 days enhances off-season production and crop intensification opportunities under changing climate regimes, allowing farmers to maximize land use and diversify sources of income (Bisht et al., 2005). By simultaneously raising productivity, enhancing resilience, and reducing emissions, rice beans offer vulnerable smallholder systems an underutilized but strategically significant path to food security and sustainable development (Andersen, 2014).

### 3.2. Adaptation to marginal environments

The rice beans are ideally adapted to marginal areas, such as rain-fed hillsides, degraded soils, and drought-prone regions, where conventional crops do not flourish. The ability of rice beans to flourish in low-nutrient soils without frequent fertilizer application reduces production costs and nitrogen emissions. Its potential as a climate-smart legume for sustainable agriculture in regions with limited resources and changing environmental conditions is highlighted by its resistance to drought and poor soils as well as its adaptability to a range of agroecological zones, including hill slopes, plateaus, and rainfed environments (Odeku et al., 2024).

### 3.3. Reduced dependence on agrochemicals

Compared to high-input commercial crops, *V. umbellata* are less reliant on pesticides and the associated ecological risks because they naturally resist major pests and diseases (Katoch, 2020). Its strong compatibility with organic and a variety of low-input farming systems further enhances its role as a climate-smart crop (Padulosi et al., 2013). Moreover, its life cycle greenhouse gas (GHG) emissions are significantly lower than those of cereals or conventional legumes because it needs less water, matures faster, and fixes nitrogen biologically. By reducing reliance on synthetic nitrogen fertilizers and thriving in intercropping or agroforestry systems, rice beans contribute to carbon sequestration and system-level sustainability, increasing their potential in low-carbon agriculture (FAO, 2013).

### 3.4. Short growth duration and drought resilience

Because they mature quickly (60–90 days), rice beans provide a short-duration, quick-return option after large harvests that is ideal for incorporating into residual or relay cropping systems (Tomooka et al., 2012). Its potential for integration is further increased by its strong compatibility with low-input diversified farming techniques like inter-cropping and agroforestry, which improve resource-use efficiency and system resilience (Stagnari et al., 2017). Because of their deep root system and natural physiological resistance to drought, rice beans flourish in regions with erratic rainfall patterns, particularly in South and Southeast Asia (Joshi et al., 2007). It demonstrates strong sustainability potential as a climate-smart pulse when compared with traditional legumes such as chickpea, pigeon pea, and common bean. It is generally associated with a lower water footprint due to its superior adaptability to rainfed, marginal, and low-input agro-ecological systems, requiring minimal supplemental irrigation. In contrast, many conventional pulse crops exhibit higher water demand and greater sensitivity to water stress during critical growth stages. This inherent water-use efficiency, combined with drought tolerance and resilience in nutrient-poor soils, positions rice bean as a strategically important crop for sustainable agriculture. Incorporating water footprint metrics reinforces its role in climate-resilient cropping systems and sustainable food security frameworks (Kumar et al., 2019). These adaptable traits make rice beans a climate-resilient strategic crop for smallholders in delicate agroecosystems.

### 3.5. Rice beans promote agro-biodiversity

Reintroducing diverse cropping systems into modern agriculture reduces dependence on a few key crops and helps to mitigate the vulnerabilities associated with monoculture, particularly in the face of stress and climate variability (Katoch, 2020; Padulosi et al., 2013). Rice beans, an adaptable and resilient crop, contribute significantly to sustainable farming systems and are in line with the principles of climate-smart agriculture by increasing productivity, ecological balance, and resilience. Furthermore, due to its ability to grow well in shaded areas, rice beans can be used as an understory legume in agro-forestry, where they improve soil and provide ecological services that enhance system diversity and sustainability (Asghar et al., 2001). Rice beans are unique in that they can fix nitrogen through symbiotic relationships with Rhizobium species in their root nodules. Particularly in farming contexts with limited resources, this biological nitrogen fixation improves soil fertility, lessens reliance on synthetic nitrogen fertilizers, and increases the sustainability of cereal-based cropping systems (Zahran, 1999).

Combined, these traits make rice beans a strategic climate-resilient legume that boosts productivity in diverse and sustainable farming systems while sustaining ecosystem services vital to agroecological stability. As the rice beans improve soil fertility, reduce reliance on external inputs, and support integrated crop livestock systems, they best exemplify the multifunctional role of underutilized legumes in advancing global agendas for food security, climate adaptation, and sustainable agriculture.

## 4. Geographical distribution and genetic diversity

### 4.1. Geographical distribution and conservation significance

Native to South and Southeast Asia, the rice bean (*V. umbellata*) has a broad, but dispersed distribution that includes both traditional cultivation centers and more recent introductions. With a natural occurrence in India and central China, its main range is in the Indo-China region, which includes Thailand, Myanmar, Laos and Vietnam. South Asia, the Chota Nagpur plateau, the northeastern Indian states of Uttarakhand and Himachal Pradesh, as well as Bangladesh Sri Lanka, and Nepal represent the main locations for cultivation (Gayacharan et al., 2024a). While rice beans are grown in southern China, Taiwan, and Korea in East Asia, they are also grown in Myanmar, Thailand Laos, Vietnam, Cambodia, Indonesia, Malaysia, and East Timor in Southeast Asia. In addition to Asia, the species has been brought to the Americas Jamaica, Haiti, Mexico the West Indies, and Hawaii in Africa, it has been brought to Ghana, Tanzania the Democratic Republic of the Congo, Sierra Leone and Fiji and in the Pacific to Ghana, Tanzania and Fiji, and Queensland Australia (Francis et al., 2023). Additionally, these mapping-driven frameworks help farmers maintain and enhance locally adapted land-races by bolstering participatory breeding programs (Jarvis et al., 2011). The cultural and nutritional significance of this underappreciated legume is preserved by these integrative conservation strategies, which also increase climate change resilience and protect agro-biodiversity.

### 4.2. Genetic diversity and breeding resources

The genetic diversity of *V. umbellata* is greatly influenced by its broad geographic range, which includes several landraces and wild relatives that have been preserved throughout Southeast and East Asia. These landraces are a storehouse of adaptive traits against climate-specific stressors such as soil acidity, cold pests, and drought. For instance, accessions from high-altitude Nepal exhibit cold tolerance, allowing for adaptation to temperate and sub-temperate agroecosystems, while Northeast Indian accessions exhibit resistance to bruchid pests and tolerance for acidic soils (Darai et al., 2023). There are opportunities to match breeding with consumer preferences and productivity goals because Southeast Asian accessions, particularly those from Thailand and Laos, exhibit notable variation in seed color, size, and yield-related traits (Tian et al., 2013). The richness of rice bean germplasm is confirmed by extensive diversity assessments. Out of 472 accessions from 16 Asian countries, 168 alleles were found, with cultivated populations from Vietnam, Myanmar, Nepal, and India showing particularly high gene diversity. However, formal breeding programs are still lacking in spite of this diversity, which means farmers are mostly dependent on traditional landraces, which is a significant barrier to systematic crop improvement (Darai et al., 2023). This genetic reservoir is vital for future breeding efforts aimed at enhancing yield, resilience, and nutritional quality under climate variability. Gene banks, including ICAR-NBPGR in India, repositories in Thailand, and the National Institute of Agrobiological Sciences in Japan, safeguard thousands of accessions to ensure germplasm availability for crop research and breeding (Gore et al., 2022). Advances in molecular characterization using Random Amplified Polymorphic DNA (RAPD), By bridging the gap between traditional landrace cultivation and modern improvement strategies, rice beans can contribute significantly to global food security, sustainable agriculture, and livelihood resilience, particularly in regions most vulnerable to climate change.

### 4.3. Threats to genetic erosion and conservation priorities

The rice bean, despite its resilience and nutritional promise, faces threats of genetic erosion. One of the main drivers is landrace replacement, as traditional varieties are increasingly substituted with high-yielding or market-preferred crops such as common bean (*Phaseolus vulgaris*) and cowpea (*Vigna unguiculata*), leading to the loss of unique allelic diversity shaped by long-term adaptation to diverse agro-ecological and cultural contexts (Pattanayak et al., 2019). Equally critical is the erosion of traditional seed systems, historically maintained by women and community networks through selection, exchange, and local propagation of diverse landraces. These informal systems are rapidly declining with the expansion of formal seed markets and commercial crop choices, resulting in both genetic and cultural losses (Tian et al., 2013; Khoury et al., 2022). In marginal environments where rice bean cultivation persists, it is often relegated to small-scale intercropping or household gardens, leaving populations vulnerable to stochastic extinction, low regeneration, and genetic bottlenecks (Andersen, 2014). Conservation priorities should therefore emphasize *in situ* strategies, including participatory varietal selection, community seed banks, and integration into climate-smart agroecological practices (Nair et al., 2023). At the same time, *ex situ* conservation, such as the inclusion of diverse *V. umbellata* accessions in repositories like ICRISAT and India’s National Bureau of Plant Genetic Resources (NBPGR), is vital for securing rare alleles and expanding the breeding base (Jha et al., 2024).

### 4.4. Modern genomics and metabolomics, proteomics insights

In order to meet the specific requirements of smallholder farmers and diverse agroecologies, modern, science-based breeding techniques have replaced traditional selection. Integrating molecular tools, farmer-centered knowledge systems, and naturally occurring and induced genetic variation is essential to these approaches. This change is best illustrated by participatory plant breeding (PPB), which encourages direct cooperation between breeders and farmers to assess genotypes in authentic on-farm settings. PPB has been especially helpful in underutilized crops like rice beans, where local and cultural contexts significantly influence trait preferences like early maturity, drought tolerance, and seed quality (Weltzien and Christinck, 2017). Marker-assisted selection (MAS), which has emerged as a vital technique for accurately monitoring and introducing genes associated with abiotic stress tolerance, disease resistance, and yield stability, has been added to PPB. Molecular markers like SSRs, RAPDs, and SNPs have been created in *Vigna* species, including rice beans, to expedite breeding pipelines (Isemura et al., 2010). Recent advances in genomics have facilitated the mapping of quantitative trait loci (QTLs) that govern critical traits such as drought resistance and seed characteristics, opening the door to genomic selection and targeted improvement (Tian et al., 2013). Methods like mutation breeding, which use physical agents like gamma radiation or chemical mutagens like EMS to create novel variability, provide ways to increase diversity by broadening the genetic base (Andersen, 2014). In a similar vein, polycrossing, which involves carefully regulated intercrosses between several parents, promotes variability in segregating populations and permits recombination of advantageous alleles (Wang et al., 2014). Due to their extreme sensitivity to changes in temperature, rainfall, and photoperiod, rice beans require a move toward breeding methods tailored to agroecology. Significant obstacles to widespread adoption include major diseases like rust (*Uromyces appendiculatus*), powdery mildew (*Oidiopsis taurica*), rhizoctonia blight (*Rhizoctonia solani*), bacterial blight (*Pseudomonas* spp.), anthracnose (*Colletotrichum truncatum*), and cercospora leaf spot (*Cercospora* spp.). The need for long-lasting genetic resistance is highlighted by the limited relief offered by traditional cultural and chemical control methods. Important alleles for resistance breeding across *Vigna* species are provided by some rice bean genotypes that show promising resistance to the yellow mosaic virus (YMV). The efficiency of marker-assisted selection will be improved by the quicker identification of resistance genes and the creation of closely related molecular markers. Furthermore, precision breeding, genome editing, and the possible use of cisgenic or transgenic rice bean lines tailored for new agricultural challenges are made possible by the molecular mechanisms underlying disease resistance being clarified through gene cloning and functional genomics (Oraon et al., 2023).

#### 4.4.1 . Metabolite-guided breeding for nutritional security

Metabolite-driven selection represents an emerging paradigm in crop improvement, whereby quantitative profiles obtained through LC–MS or NMR are harnessed as phenotypic markers for nutritional, processing, and resistance-related traits. This strategy is particularly relevant for underutilized legumes, where consumer adoption depends as much on sensory and functional quality as on agronomic yield. In *V. umbellata*, emerging metabolite baselines provide a foundation for such efforts: comprehensive LC–MS fingerprints across free and bound fractions have revealed a diverse array of flavonoids and phenolic acids, profiled under multiple extraction regimes and enabling the development of compound-level marker panels (Jiang et al., 2023). Together, these baselines establish a biochemical framework for linking metabolite diversity with breeding outcomes. Beyond descriptive profiling, metabolomics is increasingly being embedded into breeding pipelines. A core LC–MS panel including phenolic acids, flavonoids, raffinose family oligosaccharides (RFOs such as raffinose and stachyose), γ-aminobutyric acid (GABA), and free amino acids, can be systematically assayed across diversity panels and early breeding generations (Jiang et al., 2023). Emerging evidence highlights robust associations between metabolite signatures and functional endpoints, including antioxidant capacity, flavor, bitterness, seed hardening, soaking and cooking time, and even pest resistance, such as correlations between seed-coat phenolics and bruchid tolerance (Li et al., 2018a).

Recent advances underscore that metabolomics also captures dynamic metabolic plasticity. Sprout-stage metabolomics, including methyl jasmonate (MeJA)-elicited germination assays, demonstrate genotype and treatment-specific shifts in flavonoid and phenylpropanoid metabolism, revealing latent biochemical flexibility exploitable for functional and value-added traits (Jiang et al., 2023). Notably, seed-coat phenolics and related metabolites correlate strongly with bruchid resistance in legumes, reinforcing the utility of metabolite-based indices that complement genetic markers (Li et al., 2019). Collectively, these findings position metabolomics not merely as a descriptive tool but as a strategic driver of breeding innovation, enabling the translation of rice bean’s biochemical richness into nutritional enhancement, pest resilience, and post-harvest stability.

Looking ahead, the integration of metabolomics with genomics and pan-genomics offers a powerful route to uncover genotype–metabolite–trait linkages at scale. High-resolution metabolite-QTL mapping, combined with transcriptomics, can resolve regulatory networks underpinning key compounds and accelerate marker-assisted or genomic selection. Future efforts should also prioritize field-level metabolomics to capture environmental modulation of metabolic traits, ensuring stability under variable agroclimatic conditions.

#### 4.4.2. Proteomics driving future rice bean breeding

Proteomics offers a critical dimension for elucidating functional effectors that underpin complex traits in *V. umbellata*. While direct proteomic studies on this legume remain scarce, work in related *Vigna* species, such as mung bean has revealed abundant seed-storage proteins, proteases, and stress-responsive enzymes, providing a foundation for candidate discovery in rice bean (Chirag et al., 2025). Beyond cataloguing proteins, integrative transcriptome proteome analyses in legumes have mapped enzyme complements within the phenylpropanoid and raffinose family oligosaccharide (RFO) pathways, which directly influence nutritional quality, anti-nutritional components, and stress resilience (Hu et al., 2025). Such datasets underscore the value of proteomics in capturing not only structural proteins but also enzymatic regulators of secondary metabolism, seed development, and environmental adaptation (Al-Obaidi et al., 2024).

Recent advances in quantitative LC–MS/MS platforms enable high-resolution protein profiling that, when coupled with transcriptomics and metabolomics, provides a systems-level perspective. This multi-omics integration is essential for validating post-transcriptional regulation, assessing enzyme activity, and establishing causal links between molecular function and phenotypic traits (Yu et al., 2020). For example, proteomic insights into RFO biosynthetic enzymes can refine breeding for reduced anti-nutritional load while maintaining stress tolerance, whereas mapping stress-inducible proteomes may reveal resilience signatures relevant to climate-smart agriculture. Importantly, proteomic markers can be embedded into breeding pipelines, complementing genomic and metabolite-based indices, to guide multi-trait selection strategies (Alrajeh et al., 2024).

Looking forward, integration of proteomics with pan-genomics and spatial metabolomics could illuminate tissue-specific and developmental dynamics, providing a future-ready framework for nutrition-sensitive and climate-resilient legume breeding.

## 5. Rice beans for food security

The *V. umbellata* is a highly nutritious but underutilized legume that has significant potential to improve food and nutrition security worldwide. Recent evidences (2025–2026) consistently highlight that climate-resilient legumes play a pivotal role in strengthening food security while simultaneously supporting rural and local economies under increasing climate stress. Climate-driven yield instability in major staples has intensified reliance on adaptive legume systems that maintain productivity under heat and water scarcity, thereby stabilizing farm-level income and dietary protein supply. Large-scale syntheses show that integrating legumes into cropping systems improves resource-use efficiency, particularly nitrogen and water use, while enhancing overall yield stability and reducing input costs, which directly benefits smallholder livelihoods and local agricultural economies. In addition, climate-resilient legumes contribute to improved food system resilience by sustaining production in marginal environments where conventional crops fail, thereby reducing economic vulnerability and import dependency in developing regions. Collectively, these findings reinforce that legumes are not only nutritional security crops but also economic stabilizers within climate-affected agrarian systems, strengthening rural resilience and market continuity under climate change pressures (Akchaya et al., 2025; Anshida et al., 2026).

An efficient plant-based protein source in malnutrition-affected areas rice beans have a balanced amino acid profile and high digestibility with 20–26 % protein mostly in the form of globulins and albumins. Its relatively low glycaemic index which results from its carbohydrate composition helps with the dietary management of diabetes and cardiometabolic disorders. Its low lipid content (1–3%) is rich in polyunsaturated fatty acids particularly linoleic and linolenic acids which support cardiovascular health and metabolic regulation (Pattanayak et al., 2019). Crucially, its lower concentration of oligosaccharides from the raffinose family reduces gastrointestinal distress and increases consumer acceptance (Katoch, 2013). Rice beans provide vital micronutrients such as iron zinc calcium magnesium and potassium in addition to macronutrients thereby addressing hidden hunger in populations with limited resources. Because they can tolerate heat and drought, grow well in marginal soils, and improve soil fertility through efficient symbiotic nitrogen fixation, rice beans are agronomically appropriate for climate-smart agriculture (Katoch et al., 2023).

Reviews and nutritional analyses highlight the potential of rice beans, but the FAO and orphan-crop literature stress the significance of markets and policy in determining scale and adoption (FAO, 2017). Policies for dietary diversification Important policy levers that can encourage public procurement and create predictable demand include school feeding programs and explicit inclusion in national food and nutrition strategies. To ensure accessibility, farmer adoption, and the quick release of locally adapted and biofortified varieties, it is equally critical to fortify seed systems like community seed networks quality-declared seed (QDS) models (Mmbando, 2025; Mabhaudhi et al., 2019). Consumer trust and market acceptance are influenced by food safety frameworks, quality labels, and standards. To successfully expand underutilized legumes and position rice beans as a beneficial climate-smart crop with a variety of socioeconomic impacts Examples include the Harvest Plus Crop Breeding Consortium and other programs (Bouis and Saltzman, 2017).

The establishment of quality labels and food safety frameworks is necessary for rice beans to realize their full market potential. Investing in small-scale processing, such as dehulling pre-cooking flours, ready-to-eat snacks, and fortified blends, can help rural businesses increase their profit margins and reduce post-harvest losses by utilizing the high protein and micronutrient profile of rice beans (Katoch et al., 2023). Simultaneously generating consumer demand through nutrition messaging, school feeding programs, maternal and child nutrition initiatives, and concentrating on urban organic or ethnic-diaspora markets is essential to promoting uptake. By providing price incentives, buy-back plans, and aggregation premiums for quality traits like bruchid resistance or biofortification targets, farmers can reduce the risk of adoption until markets stabilize. Programs for biofortified crops, such as the CBC, demonstrate this (Bouis et al., 2024).

Funding and institutional arrangements are crucial to transforming rice bean from an underutilized orphan crop into a widely cultivated and economically viable species. Public sector engagement is particularly essential, especially through national agricultural research systems (NARS), which should leverage omics-based innovations such as molecular markers and genomic selection models to accelerate variety development and integrate rice bean into mainstream pulse breeding pipelines. Recent transcriptomics and genomics studies on rice beans provide actionable targets for NARS breeding programs (Supritha et al., 2025). In addition to providing funds for consumer research to direct product development, donors and development partners can act as catalysts. So, by employing blended finance models to reduce the risk of private investments in branded product lines and processing facilities for start-up companies. Harvest Plus and CGIAR Commercialization initiatives demonstrate that strategic finance and product testing are essential for successful crop scaling (Apampa et al., 2021). With enabling policies like matching grants and tax incentives, the private sector which includes retailers, food-tech start-ups, and processors can anchor rice bean value chains by developing protein concentrates and fortified blends for convenience foods (Nordhagen and Demmler, 2023).

Reviews on NUS commercialization emphasize opportunities for value addition and small-scale processing aimed at gender and youth (Malhotra et al., 2024). Successful scaling models, particularly those from Harvest Plus and the CBC program, emphasize the need for integrated seed-to-market planning, strong aggregation systems, and continuous public-sector involvement. These lessons can have a significant impact on rice bean commercialization (Apampa et al., 2021). FAO’s NUS initiatives provide policy templates and capacity-building strategies to integrate rice beans into national agricultural agendas and diet-diversification programs (Bogliotti et al., 2021).

Strong metrics are needed to create a valid link between rice bean science and nutritional and socioeconomic outcomes. Important metrics include market margins, post-harvest losses, processing capacity, and aggregation volumes and adoption rates of different varieties. Regular monitoring of nutrition outcomes such as improved dietary diversity and micronutrient intake (iron, zinc, and protein quality), as well as economic and environmental benefits, is necessary (Borelli et al., 2020). Rice beans can be guaranteed to fulfill their potential for enhancing livelihoods and guaranteeing nutrition security by combining scientific discoveries with deliberate demand generation aggregation and enabling policies, as demonstrated by experiments with models like Harvest Plus CBC and FAO’s NUS frameworks (Xu et al., 2022).

## 6. Ethnobotanical and cultural significance

### 6.1. Traditional culinary and medicinal uses

Asides from rice bean tender pods valued as vegetables, the grains are used to make traditional dishes like dhal soups and gruels. Beyond staples, rice beans’ versatility and cultural embeddedness are demonstrated by their use in a wide range of culinary preparations, including pickles, pastries, sweets, and snacks (Ahmed and Jamil, 2024).

The rice bean has important ethnobotanical and cultural significance throughout its natural range in South and Southeast Asia. Seasonal customs, medicinal practices, and regional cuisines all heavily incorporate it. In Nepal, rice beans play a crucial role in Kwati, a mixed-legume soup that is customarily consumed during the Janai Purnima festival and symbolizes both sustenance and ceremonial health. Rice beans are a centerpiece of Sepu Vadi, a festive dish in Himachal Pradesh, India, which highlights how deeply embedded they are in regional culinary traditions. Rice beans have long been valued for their culinary properties in traditional medical systems. For instance, they are utilized as an anti-inflammatory and digestive tonic in Indian and Nepalese traditional medicine (Ahmed and Jamil, 2024). As the seeds exhibit a rich profile of phenolics and flavonoids i.e., vitexin, catechin, isovitexin, quercetin, gallic acid, and rutin, which confer antioxidant, anti-inflammatory, and antimicrobial activities, phytochemical evidence supporting its ethnopharmacological relevance is growing (Jiang et al., 2023; Li et al., 2018a). By maintaining gut homeostasis, enhancing immunological responses, and lowering oxidative stress, these bioactive compounds are thought to close the gap between accepted knowledge and recent biomedical discoveries.

Traditionally, seeds are consumed as pulses with rice or boiled and added to soups. In East Asia, which includes China, Japan, and Korea, rice beans are frequently ground into meal or flour and used in baked goods and porridge. Despite these regional preferences, rice beans continue to provide dietary diversity despite their strong flavor and potential for flatulence, which remain barriers to wider acceptance (Gupta et al., 2013). There are numerous ethnobotanical applications for rice beans outside of the kitchen. Its leaves, sprouts, and processed forms highlight its dual role in sustenance and health by demonstrating how it can be used as a staple food or as a functional dietary component. Documenting these traditions is crucial for preserving the intangible cultural heritage embedded in agro-food systems, in addition to safe-guarding genetic diversity. In an era of increasing crop marginalization and dietary homogenization, it is imperative to increase awareness of the cultural and nutritional significance of rice beans. More research on this underutilized legume may enhance its contribution to dietary diversity, food security, and long-term health solutions in regions where malnutrition is an issue (Atta et al., 2022).

### 6.2. Socio-economic relevance in rural communities

Throughout South and Southeast Asia rice beans are an underestimated, but economically and nutritionally significant legume in rural and tribal communities. It has long been grown in upland marginal and hill ecosystems such as those in Nepal northeastern India and Southeast Asia. Additionally, it supports food security and dietary diversity and stabilizes livelihoods in fragile agroecosystems (Dahipahle et al., 2017). The species is a low-cost climate adaptation strategy for smallholder farmers navigating uncertain environments because of its resilience to abiotic stresses and its multipurpose value as green manure, livestock feed, erosion control, and human nutrition. Although digestibility is limited by antinutritional factors, rice bean grains are high in protein, dietary fiber, essential amino acids, and micronutrients. To lessen these limitations, traditional processing techniques like dehulling, soaking, boiling, sprouting, and fermentation are frequently used. These treatments increase the bioavailability of minerals and amino acids, decrease tannins and phytic acid, and denature antinutritional proteins (Dhillon and Tanwar, 2018).

Despite these advantages, other pulses such as cowpea, mung, and urd beans are still thought to be superior to rice beans. Insufficient supply networks It cannot be widely adopted and commercialized due to low market awareness, a lack of extension services, and a lack of private sector investment (Dhillon and Tanwar, 2018). Nonetheless, its production contributes to the preservation of agro-biodiversity, particularly through community seed systems and landrace conservation on farms. National and international gene banks safeguard a vast amount of rice bean germplasm, and new advancements in breeding and genomic resources offer opportunities to produce improved varieties better suited to the needs of farmers and consumers (Dahipahle et al., 2017). Only through integrated strategies that combine investments in market and processing infrastructure with gender-sensitive extension value chain development and participatory breeding can rice beans reach their full potential.

### 6.3. Gender dimensions in cultivation and use of rice beans

Gender has a major impact on *V. umbellata* cultivation, despite the fact that little is known about this area of legume research. Women produce rice beans at a significantly higher rate than men in Nyanza, Kenya (2.96 vs. 2.39) (George et al., 2022). Their duty as crop stewards is highlighted by the fact that women typically perform labor-intensive tasks like weeding, processing, and seed preservation. This pattern aligns with broader agricultural trends that indicate women in developing regions produce 60–80% of the food produced, despite structural disadvantages in access to extension services and agricultural inputs related to land tenure (Malkanthi, 2016). The resilience and productivity of female farmers are restricted by these systemic injustices, even though they are the primary providers of household food security (Perelli et al., 2024). Empowering women farmers not only corrects historical injustices but also enhances adoption and sustainability outcomes, making rice beans an essential crop for equitable, resilient, and inclusive food systems in the face of climate change (Bryan et al., 2024).

## 7. Nutritional composition and bioactive constituents

### 7.1. Macronutrients: protein, fat, carbohydrates, dietary fiber

**Fig. 2.**
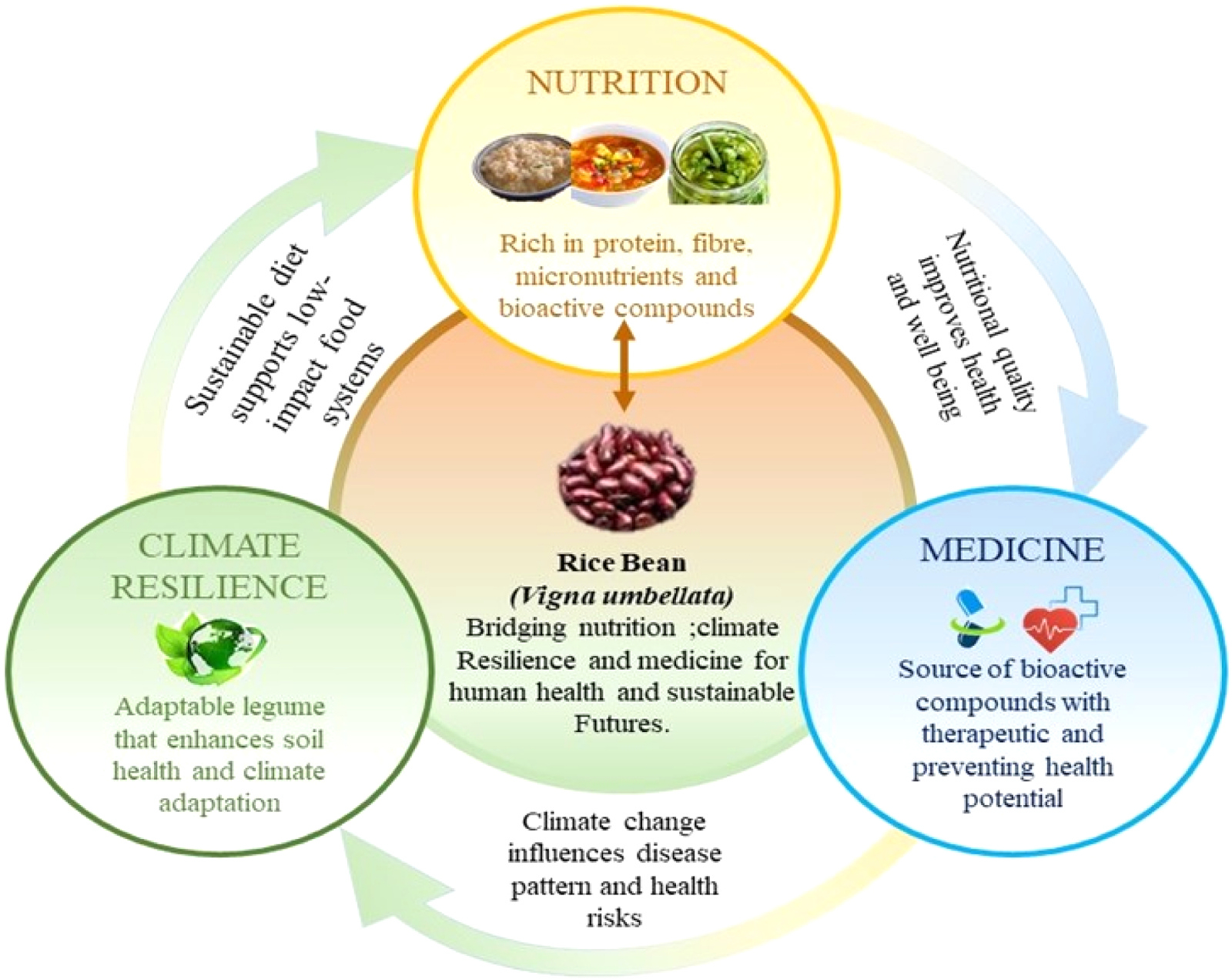
Schematic diagram showing significance of rice bean *(Vigna umbellata)* and its interdisciplinary relationship with medicine, climate resilience and nutrition.

Because of its high protein content and beneficial macronutrient profile, rice bean (*V. umbellata*) is increasingly recognized as a nutrient-dense but underutilized legume (Fig. 2). Agronomic practices and genotype protein levels are said to vary between 17 and 25 %, depending on environmental factors. For example, the BRS–2 genotype can reach up to 25.6% in vitro and has an approximate digestibility of 54%. Comparative studies revealed that seed protein content of rice beans varies between 17.3% and 21.4%, closely matching that of popular legumes like mung beans (*V. radiata*) and cowpeas (*V. unguiculata*), highlight its significance for dietary protein supplementation (Kaur et al., 2013). As is typical for pulse crops, rice bean has relatively low-fat levels (an average of 3.5–4.0%), and their macronutrient composition is dominated by carbohydrates (~61–65%), making them an energy-dense food source for both functional and subsistence diets. By improving bowel function and controlling microbiota, crude fiber, which accounts for 5–7% of seed weight, also aids in dietary fiber intake and supports gut health (Katoch, 2013). At the protein fraction level, rice beans have a similar profile to other legumes, with glutelins and prolamins making up a smaller portion and albumins and globulins acting as the primary storage proteins. About 11–15% globulins and 6–7% albumins make up the primary protein reservoir, which affects both nutritional quality and functional food applications (Rodriguez and Mendoza, 1991). *V. umbellata* offers a nutritionally balanced amino acid profile, with ~40% of its protein fraction comprising essential amino acids (EAAs). Non-essential amino acids (NEAAs) such as aspartic acid, glutamic acid, serine, glycine, alanine, proline, and arginine further enrich its proteome (Katoch et al., 2023; Yao et al., 2012). Notably, it’s high lysine content often limiting in cereals enhances the nutritional complementarity of mixed diets. Beyond basal nutrition, rice bean proteins are a source of bioactive peptides with diverse health-promoting functions. Enzymatic hydrolysis, particularly with alcalase, yields peptides that inhibit angiotensin-I converting enzyme (ACE), exerting antihypertensive activity (Wang et al., 2014). Hydrolyzed peptides also display robust antioxidant potential by scavenging free radicals (DPPH, ABTS), suppressing lipid peroxidation, and chelating transition metals (González-Cruz et al., 2025). Immunomodulatory roles have also been described, with peptides stimulating macrophage activity and immune pathways, underscoring the nutraceutical promise of rice bean proteins (Hoque Bepary et al., 2016). Comparative analysis reveals distinct contrasts across legumes. Soybean (*Glycine max*), with 35–40% seed protein, provides both EAAs and NEAAs, though sulfur amino acids (methionine, cysteine) are limiting, contributing only 2–3 g/100 g protein relative to the FAO/WHO reference (Tan et al., 2023). Mung bean (*V. radiata*), contributing 20–28% protein, is enriched in EAAs such as leucine, lysine, isoleucine, valine, phenylalanine, and threonine, while glutamic and aspartic acids dominate its NEAA fraction, enhancing solubility, umami taste, and precursor functions (Li et al., 2023). Black gram (*V. mungo*) protein is similarly dominated by acidic amino acids glutamic acid (14.16–14.99 g/100 g protein) and aspartic acid (13.09–13.44 g/100 g protein) with leucine (~7.0 g/100 g protein) as the most abundant EAA, though sulfur amino acids remain limiting. Processing, including soaking, boiling, and roasting, further modulates amino acid composition, with roasting particularly enhancing EAA levels and nutritional value (Suneja et al., 2011). This hybrid nutritional and functional profile highlights rice bean as a promising dietary and nutraceutical legume.

**Fig. 3.**
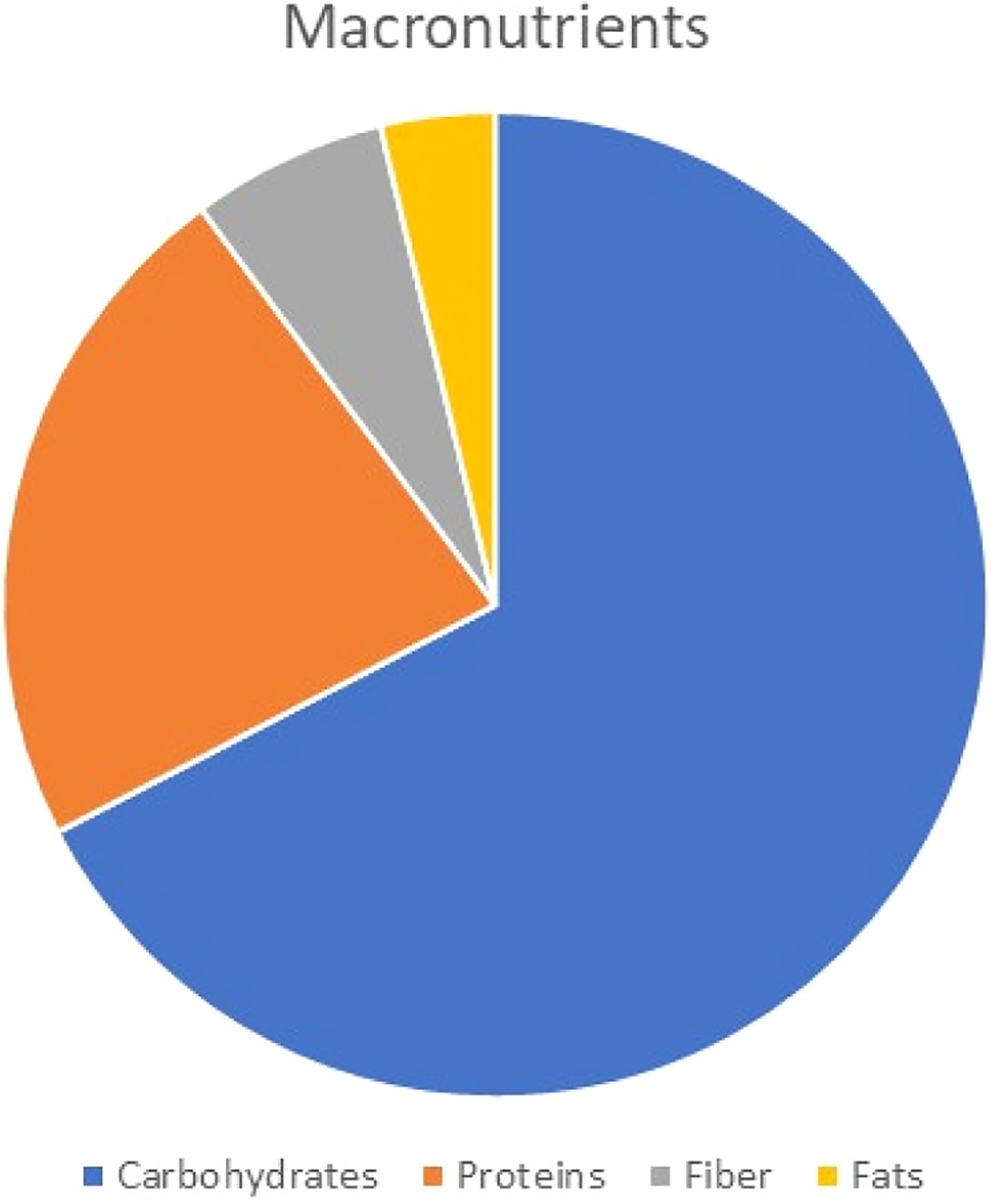
Pie chart showing distribution of micronutrients of macronutrients in rice bean (*Vigna umbellata*). Carbohydrates (CHO): 61–65%, Proteins (PRO): 17–25%, Fiber (DF): 5–7%, Fats(FAT): 3.5–4%.

*In-vitro* protein digestibility (IVPD) of rice beans has been reported to vary relatively widely, ranging from 53 to 57% percent across different accessions. More comprehensive protein fraction studies have revealed noticeably higher digestibility values; the digestibility of seed meal has been reported to be between 82 and 86 %. globulins to show digestibility between 76 and 83 %, and albumins to reach 86–88 % (Orlien et al., 2023). This suggests that some rice bean protein fractions may have higher bioavailability than the crude seed protein profile. Processing interventions have a significant impact on the modulation of protein quality and overall nutritional value. Thermal processing methods such as boiling and roasting have been shown to enhance protein digestibility by reducing the activity of antinutritional factors, in addition to denaturing structural proteins. For instance, it has been shown that heat treatment of rice bean protein greatly increases its digestibility and availability of amino acids, raising its Relative Nutritive Value (RNV) to roughly 79 %. As demonstrated by the variability across studies, processing is crucial to overcoming natural antinutritional barriers such as tannins, trypsin inhibitors, and phytic acid, which otherwise impair proteolytic enzyme activity and mineral bioavailability (Katoch et al., 2023). Mitigating these factors using biotechnological interventions and optimized processing is essential to positioning rice beans as a viable source of protein for animal and human nutrition (Fig. 3).

### 7.2. Micronutrients

**Fig. 4.**
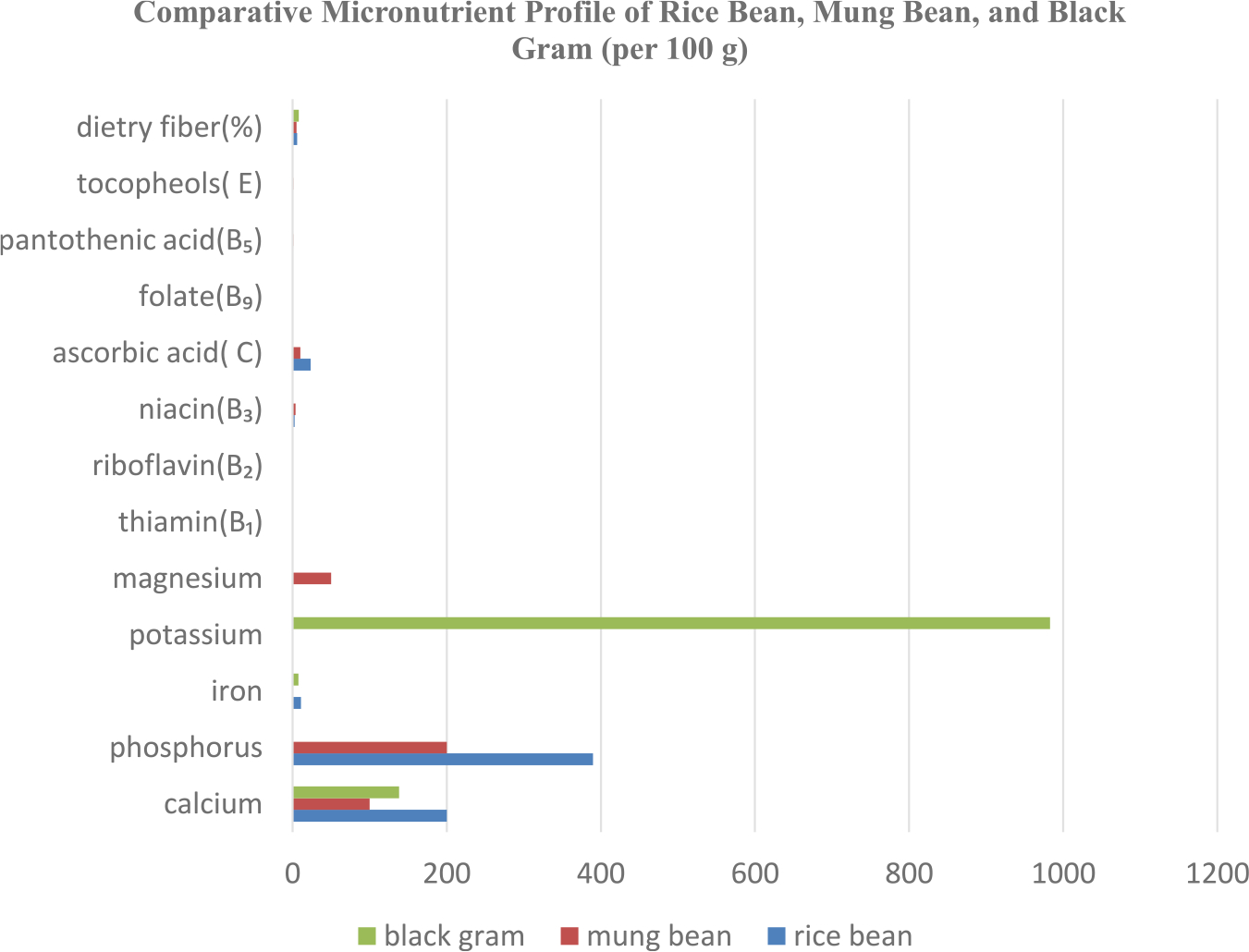
Comparative variability of minerals and vitamins composition among selected genotypes of Rice bean *(Vigna umbellata)*. Mineral include calcium (Ca), phosphorus(Ph), iron (Fe), and potassium(K); vitamins include vitamin C (ascorbic acid, AA), niacin(B3) and others.

Its micronutrient profile is equally compelling (: niacin levels reach up to 3.87 mg/100 g and ascorbic acid up to 23.46 mg/100 g, reflecting strong genotype-dependent variability (Katoch et al., 2023). By comparison, mung bean (*V. radiata*) provides a broad spectrum of vitamins, thiamine, riboflavin, pantothenic acid, vitamin C, niacin, and tocopherols, alongside calcium, magnesium, phosphorus, potassium, and trace elements essential for metabolic and skeletal health (Mehta et al., 2021). Black gram (*V. mungo*) further enriches the legume landscape with high potassium (~983 mg/100 g), calcium (~138 mg), iron (~7.6 mg), folate (~628 μg), and cultivar-specific elevations in vitamin B_2_ and dietary fiber (Sahasakul et al., 2022). When positioned within comparative phytochemical mapping, rice bean stands out through its balanced phenolic acids, structurally diverse flavonoids, and amino acid complement, making it a distinctive source of micronutrient-linked phytochemicals (Tables 1 and 2). The mineral concentrations per 100 g dry seeds, which include calcium (~200 mg range 68–230 mg), phosphorus (~390 mg range 209–370 mg), and iron (~10.9 mg), highlight variation across genotypes and environments. The following vitamins are noteworthy: thiamin (B_1_) *ca*. 0.49 mg (0. 5–1. 09 mg), riboflavin (B_2_) *ca*. 0.21 mg (0. 18–0. 5 mg), and niacin (B_3_) *ca*. 2.4 mg (2. 0–3. 6 mg). Rice beans are positioned as a promising dietary legume for addressing micronutrient deficiencies in susceptible groups due to their combined capacity to support oxidative metabolism, hemopoiesis, and neurological health. The substantial variation observed across various studies underscores the necessity of optimizing breeding programs by genotype-by-environment and illustrates the potent influence of genetic diversity (Fig. 4), soil nutrient status, and agroclimatic conditions on mineral and vitamin composition (Gayacharan et al., 2024b).

### 7.3. Bioactives

Comparative profiling provides valuable information about species-specific potential, and the phytochemical composition of legumes determines their nutritional and medicinal value (Table 3). A legume’s potential for use in nutraceuticals is highlighted by variation in phytochemical composition of rice bean (*V. umbellata*), which both resembles and differs from that of mung bean (*V. radiata*), black gram (*V. mungo*), and soybean (*Glycine max*). *V. umbellata* exhibits a distinctive polyphenolic fingerprint shaped by genotype and environment, with total phenolic content ranging between 2–5 mg GAE/g (Yao et al., 2012). Its phytochemical spectrum is characterized by abundant phenolic acids, gallic, caffeic, ferulic, and p-coumaric, which collectively contribute to antioxidant and metabolic resilience (Katoch, 2013). This profile overlaps with black gram (*V. mungo*), which also contains vanillic and protocatechuic acids, thereby broadening its functional diversity. Gallic (phenolic acid), caffeic and ferulic (hydroxycinnamic acids), vanillic (benzoic acid derivative), protocatechuic (dihydroxybenzoic acid), and p-coumaric (hydroxycinnamic acid) acids are found in considerable amounts in *V. mungo* (black gram), while *V. radiata* (mung bean) accumulates more protocatechuic and chlorogenic acids. By contrast, mung bean (*V. radiata*) specializes in protocatechuic and chlorogenic acids, conferring unique antioxidant potential, while soybean (*Glycine max*) diverges with dominance of isoflavones genistein, daidzein, and glycitein, that define its distinct hormonal and metabolic activities (Kaur et al., 2022).

The flavonoid architecture of rice bean further distinguishes it among legumes. It is enriched in quercetin, rutin, isoquercitrin, kaempferol–3-O-rutinoside, and the dihydroflavonol taxifolin, alongside flavan–3-ols such as catechin and procyanidin B1 (Jiang et al., 2023). In black gram, quercetin, kaempferol, rutin, apigenin, luteolin, and vitexin dominate (Gonzàlez-Cruz et al., 2025), whereas mung bean is defined by its C-glycosyl flavones—orientin, isoorientin, vitexin, and isovitexin with seed and seed coats accumulating vitexin (~130.5 ± 17.9 mg/g) and isovitexin (~21.2 ± 3.2 mg/g) in freeze-dried extracts, substantially exceeding water-based preparations (Tan et al., 2023). Soybean, in contrast, remains isoflavone-rich, with genistein, daidzein, and glycitein (5–10%) as its hallmark, while other flavonols like kaempferol, quercetin, rutin, luteolin, and apigenin are largely confined to seed coats and foliage (Fan et al., 2025). Taken together, rice bean emerges as a nutraceutically unique legume: it is devoid of isoflavones yet abundant in flavan–3-ols, dihydroflavonols, and flavonol glycosides. Unlike mung bean, it lacks C-glycosyl flavones. Unlike soybean, it avoids phytoestrogen dominance. Its hybrid signature, rutin, isoquercitrin, catechin, procyanidin B1, and taxifolin, positions rice bean as a functionally versatile and metabolically resilient legume with translational potential in dietary and therapeutic contexts. The rice beans present a rich source of bioactive phenolics and proteins with notable nutraceutical potential. Total phenolic content ranges between 1.6–1.8% dry weight, with tannins (~1.4–1.6%) and additional anti-nutritional factors such as trypsin inhibitors, phytic acid, and saponins contributing to its complex chemical composition (Ahmed and Jamil, 2024; Katoch, 2013). Bound phenolics dominate (~71% of total; 14.18 mg GAE/g), with base hydrolysis proving most effective in their release. Free phenolics include isoquercitrin, procyanidin B_1_, rutin, taxifolin, and catechin, whereas the bound fraction comprises gallic acid, protocatechuic acid, p-hydroxybenzoic acid, catechin, and phloroglucinol, the latter exhibiting superior antioxidant capacity (Jiang et al., 2023). Beyond phenolics, protein-derived peptides exert anti-fungal activity against *Botrytis cinerea*, *Fusarium oxysporum*, and *Rhizoctonia solani*, alongside mitogenic and anti-HIV–1 reverse transcriptase effects (Rajerison, 2006). Simulated gastrointestinal hydroly-sates enhance phenolic release, antioxidant activity, and exhibit antiproliferative effects against MDA (breast) and SiHa (cervical) cancer cell lines (IC_50_ ~1000 μg/mL) (Gonzàlez-Cruz et al., 2025). Additional pharmacological activities include inhibition of α-glucosidase, dipeptidyl peptidase, and hepatoprotection against acetaminophen-induced toxicity (Table 3) (Jiang et al., 2023).

## 8. Phytochemical profile and regional variation

The phytochemical composition of rice beans varies widely and is influenced by agroecological factors such as soil type, elevation, rainfall, and ambient temperature (Fig. 4). These environmental factors control the biosynthesis and accumulation of important metabolites, including phenolic acids, flavonoids, including quercetin and catechin, and different antioxidant compounds. Comparative studies of rice bean germplasm from China, India, Nepal, and Thailand demonstrate significant differences in polyphenol concentrations and antioxidant potential, highlighting the interaction between genetic and environmental factors (Iangrai et al., 2017). This phytochemical heterogeneity directly affects nutritional value, particularly protein iron and zinc levels, as well as pharmacological traits like antidiabetic and antioxidant activity (Rani and Khabiruddin, 2017). Furthermore, these biochemical changes impact sensory and processing attributes like texture, palatability, and cooking time all of which are critical in determining consumer acceptance and food innovation. Clarifying this biochemical diversity is essential for breeding plans, germplasm conservation, and expanding the potential of rice beans as a nutrient-dense and therapeutic legume Table 4).

### 8.1. Southern China

The primary determinants of the notable phytochemical variability of the *V. umbellata* across geographic regions are soil composition and climate altitude landrace genetics. Flavonoids and phenolic acids, particularly quercetin epicatechin catechin ferulic acid and sinapic acid, which are particularly prevalent in southern Chinese accessions, support its antioxidant and antidiabetic qualities. A comparative analysis of 13 Chinese cultivars revealed significant variations in flavonoid concentrations (55. 9–320. 4 mg catechin equivalents/g) and total phenolic content (123. 1–843. 8 μg/g) with UPLC-MS verifying the existence of a variety of bioactive compounds. Significant antidiabetic efficacy was highlighted by the strong correlation between these phytochemical differences and functional properties such as increased antioxidant activity α-glucosidase inhibition (44. 3–68. 7%) and suppression of advanced glycation end-products (34. 1–75. 8%). When taken as a whole these results show that the nutritional and medicinal value of rice beans is closely related to their place of origin highlighting the significance of region-specific germplasm in promoting the development of functional foods nutraceutical innovation and crop improvement techniques focused on metabolic health (Yao et al., 2012).

### 8.2. Northeast India (Nagaland, Manipur, Assam)

There are notable regional variations in the phytochemical and nutritional composition of *V. umbellata*. Cold-humid agroecology has shaped the distinctive traits of the landraces of northeast India, including Nagaland, Manipur, and Assam. The Himalayan belt encourages the accumulation of stress-sensitive metabolites, which raises protein levels and produces unique flavone glycosides with immunomodulatory, anti-inflammatory, and antioxidant properties, such as isovitexin and vitexin. These landraces enrich the albumin and globulin fractions, increasing their nutritional value and potential for food security. High altitude and conventional soil systems under ecological stress appear to encourage the biosynthesis of secondary metabolites, which enhances antioxidant capacity and adaptive functional quality. The Northeast Indian rice bean germplasm’s nutraceutical value and potential for producing functional foods with high protein content and particular health benefits are highlighted by this region-specific variation taken together (Hoque Bepary et al., 2016).

### 8.3. Mid-hill Nepal

Traditional farming practices and altitude-driven agroecological factors contribute to the distinct bioactive profile of rice beans landraces from Nepal’s mid-hill regions. Their seasonal use as a warming food is consistent with a thermogenic phytochemical composition that is augmented by environmental stressors. Higher concentrations of tannins, phytic acid, and phenolic acids, particularly ferulic and sinapic acid define these accessions. These compounds enhance antioxidant capacity and promote mineral chelation (Table 1). Additionally, protein fractions generate useful peptides like trypsin and chymotrypsin inhibitors, which alter enzymes and may be beneficial for digestion, glycemic control, and cardiovascular health. These traits all demonstrate the nutritional and cultural significance of Nepalese rice bean germ-plasm (Andersen and Chandyo, 2010).

### 8.4. Thailand and Laos

*V. umbellata* landraces from Thailand and Laos have highly pigmented seed coats that vary in color from purple and brown to mottled patterns, which indicates a dense accumulation of phenolic compounds, particularly tannins and anthocyanins. Tannins, a common component of pigmented coats, have antimutagenic and anti-carcinogenic qualities by slowing the growth of tumor cells and changing apoptosis and cell signaling pathways. They also increase antioxidant capacity generally. Collectively, these traits demonstrate the therapeutic and nutraceutical potential of Thai and Lao rice bean germplasm, highlighting their importance in influencing future programs aimed at promoting dietary health and preventing chronic diseases (Isemura et al., 2010).

### 8.5. Japan (Imported varieties)

Japanese rice beans imports are notable for their advantageous nutritional profile, particularly their resistance to starch and isoflavone enrichment (Table 1). Moderate levels of isoflavones, a class of phytoestrogenic compounds with a number of health-promoting qualities like hormone-modulating, cardioprotective, and antioxidant effects, are present in these accessions. In addition to this resistant starch, a non-digestible carbohydrate that ferments in the colon and resists enzymatic hydrolysis in the small intestine has a low glycemic index (GI). This process lowers postprandial glucose excursions, improves gut health by generating short-chain fatty acids, and increases insulin sensitivity. The dual presence of resistant starch and isoflavones in imported Japanese rice beans highlights their nutraceutical potential and supports their inclusion in dietary strategies to prevent metabolic syndrome and cardiovascular disease (Rodriguez and Mendoza, 1991).

## 9. Anti-nutritional factors and nutrient bioavailability

### 9.1. Anti-nutritional factors and their implications

Like most grain legumes, rice bean (*V. umbellata*) contains a spectrum of anti-nutritional factors, primarily phytates, tannins, and protease inhibitors, which collectively influence nutrient utilization and bioavailability. Although these constituents are intrinsic components of the seed matrix and serve ecological roles in plant defense and phosphorus storage, they impose significant constraints on the nutritional efficiency of minerals, proteins, and associated bioactive compounds. Consequently, the nutritional value of rice bean cannot be accurately assessed solely on compositional abundance, but must be interpreted in relation to anti-nutrient burden and mineral bioaccessibility (Salim et al., 2023).

Phytic acid (myo-inositol hexakisphosphate) is the principal storage form of phosphorus in rice bean seeds and exhibits strong chelating affinity for divalent minerals such as iron, zinc, calcium, and magnesium. The formation of insoluble phytate–mineral complexes reduces mineral solubility and intestinal absorption, particularly in diets where legumes constitute a major protein source. Variability in reported mineral content across rice bean genotypes must therefore be interpreted in conjunction with phytate levels, as high total mineral concentrations do not necessarily translate into high nutritional availability (Sahu et al., 2024a).

Tannins, particularly concentrated in the seed coat of pigmented rice bean varieties, further modulate nutritional quality. These polyphenolic compounds can bind dietary proteins and digestive enzymes, leading to reduced protein digestibility and amino acid availability. While tannins also contribute to antioxidant capacity in *in vitro* assays, their dual role as both bioactive and anti-nutritional agents complicates the interpretation of compositional and functional data. High tannin content may thus simultaneously inflate antioxidant metrics while impairing protein utilization (Okaiyeto et al., 2025).

Trypsin and chymotrypsin inhibitors represent another significant class of anti-nutritional factors in rice bean. By inhibiting pancreatic proteases, these compounds reduce protein digestibility and may induce compensatory pancreatic responses in animal models. Reported improvements in protein digestibility following processing must therefore be evaluated relative to baseline inhibitor activity, which varies with genotype, maturity, and environmental conditions (Condori and de Camargo, 2023).

### 9.2. Processing-driven mitigation strategies

A range of traditional and modern processing technologies has been employed to mitigate anti-nutritional factors and enhance the overall nutritional quality of rice bean. Soaking and germination activate endogenous seed phytases, facilitating partial hydrolysis of phytic acid and thereby improving mineral bioavailability. These processes also induce structural and chemical modifications in tannins, reducing their reactivity and protein-binding capacity. Fermentation represents one of the most effective biological strategies, wherein microbial phytases produced by lactic acid bacteria, yeasts, and filamentous fungi hydrolyze phytic acid into lower inositol phosphates, resulting in significantly improved mineral bioaccessibility. In addition, concurrent enzymatic and metabolic transformations during fermentation contribute to reductions in tannin content and enhancements in protein digestibility. Thermal processing techniques, including boiling and autoclaving, provide moderate reductions in tannins and heat-labile protease inhibitors; however, their effect on phytic acid is comparatively limited unless applied in combination with complementary treatments. Similarly, boiling and pressure cooking denature anti-nutritional proteins and improve overall protein digestibility, although their efficacy varies depending on processing intensity and seed matrix characteristics. Effective dehulling and soaking further reduce anti-nutritional load by removing seed coats and leaching water-soluble compounds such as tannins and a portion of phytic acid (Bepary et al., 2017; Kaur et al., 2020). Sprouting and fermentation further enhance nutrient bioavailability by activating endogenous enzymatic systems that degrade anti-nutrients and release bound micronutrients, thereby improving the functional nutritional profile of legumes (Abbas and Ahmad, 2018). Despite these benefits, the full nutritional potential of rice bean remains underexploited, and limited information is available regarding how different processing strategies influence its metabolome and proteome at a systems level. Among advanced technologies, extrusion processing—an emerging high-temperature short-time (HTST) technique has demonstrated high efficiency in simultaneously reducing multiple anti-nutritional factors. The combined effects of shear, pressure, and temperature disrupt seed microstructure, enhance enzyme accessibility, and promote degradation of phytate–mineral complexes as well as proteinaceous inhibitors (Sarra et al., 2026).

Overall, traditional and industrial processing methods including soaking, dehulling, thermal treatment, germination, and fermentation effectively reduce anti-nutritional factors to varying degrees; however, their efficiency in rice bean remains insufficiently quantified, particularly regarding residual phytate mineral interactions and protease inhibitor activity after processing. Furthermore, processing may differentially influence bioactive phenolics and other nutritional constituents, necessitating a balanced assessment of compositional trade-offs. From a food composition perspective, the presence of anti-nutritional factors highlights the need to integrate bioavailability metrics into nutritional evaluation frameworks. Future studies should report anti-nutrient levels alongside nutrient profiles, evaluate molar ratios such as phytate-to-mineral indices, and standardize analytical approaches to quantify processing-induced changes. Such integration is essential for accurately assessing the nutritional potential of rice bean and distinguishing compositional richness from true functional nutritional value (Salim et al., 2023).

## 10. Mechanistic and composition driven biofunctional insights

**Fig. 5.**
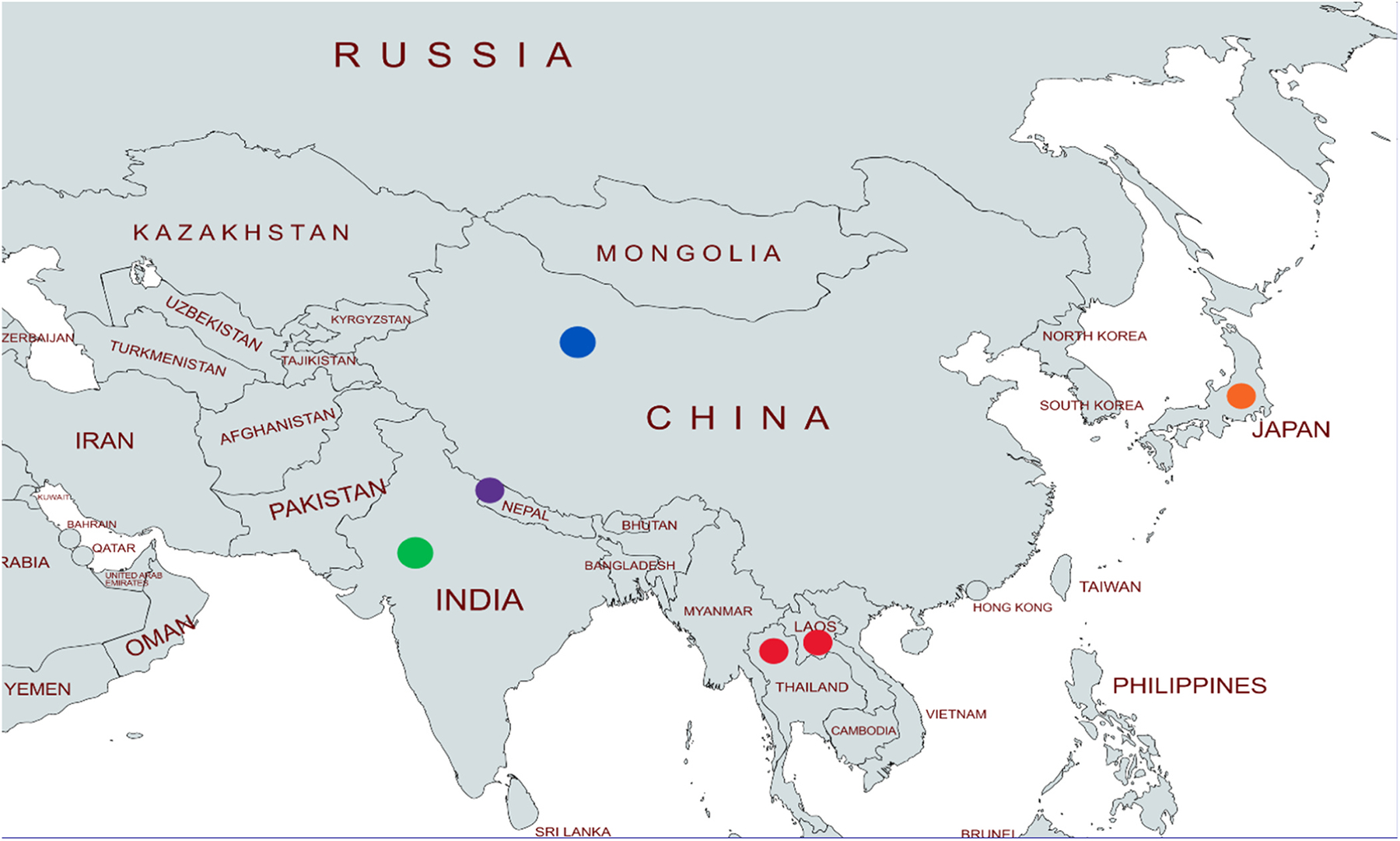
Map showing marked phytochemicals distributed in different countries of Asia and the type of nutrients present in those countries. **China**: High flavonoid & phenolic acids; **India**: Protein-rich, Isovitexin/vitexin; **Nepal**: Tannin-rich, mineral chelation; **Thailand/Laos**: Pigmented anthocyanin-rich; **Japan**: Isoflavone & resistant starch. (Map taken from web and marking done by author).

**Fig. 6.**
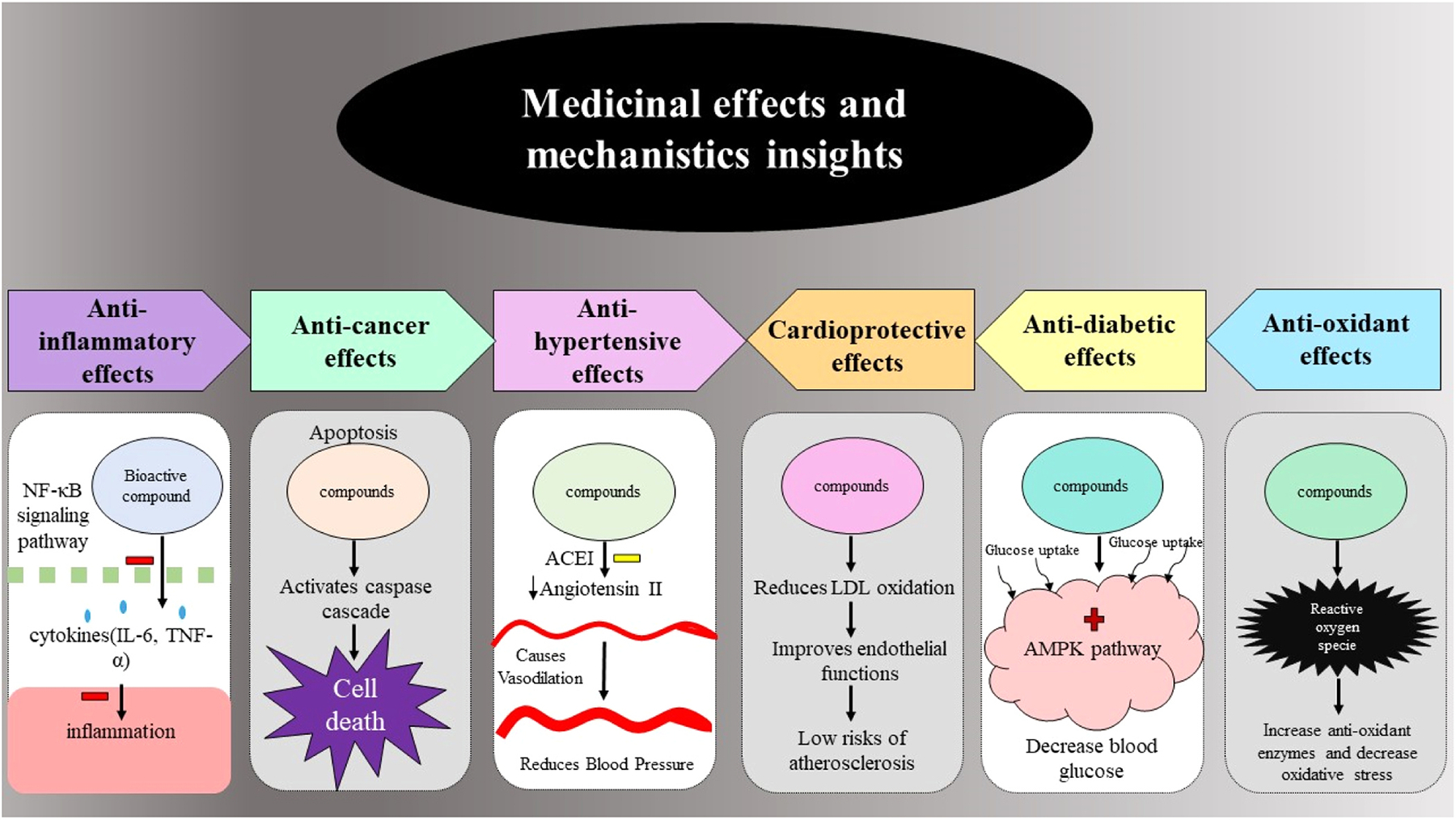
Diagrammatical representation of medicinal and health promoting effects of Rice bean (*V. umbellata*) including anti-oxidant, anti-inflammatory, anti-cancer, anti-hypertensive, cardioprotective, anti-diabetic properties.

Rice beans exhibit notable medicinal potential, with reported effects on metabolic health, cardiovascular protection, antioxidative defense, and antimicrobial activity, largely mediated by its rich phytochemical profile that are widely distributed (Figs. 5 and 6).

### 10.1. Anti-inflammatory and anticancer peptides

The rice bean is a source of high-quality protein and bioactive peptides with potential health-promoting properties. These peptides, released during gastrointestinal enzymatic digestion, have been reported to exhibit antioxidant, anti-inflammatory, and antiproliferative activities based on *in vitro* and cell-based model studies, although their precise mechanistic roles remain underexplored. Total phenolic content and antioxidant activity were also quantified, with Pearson correlation analysis performed to examine their relationship with anticancer effects. The hydrolysate demonstrated significantly higher phenolic content and antioxidant capacity than the isolate, corresponding to approximately twofold greater inhibition of cancer cell proliferation, with IC_50_ values of 1000 μg/mL after 48 h. Mechanistic insights indicate that bioactive peptides and phenolics may act via reactive oxygen species (ROS) scavenging, modulation of inflammatory pathways including NF-κB and COX–2, induction of apoptosis, and suppression of pro-inflammatory cytokines such as TNF-α and IL–6. Collectively, these findings indicate that rice bean protein hydrolysates possess mechanistic relevance inferred from compositional and preclinical studies, supporting their exploration as functional ingredients in nutraceutical and functional food formulations rather than as established therapeutic agents. Further *in vivo* investigations and detailed structure–activity analyses are required to clarify their biological relevance and translational scope (Gonzàlez-Cruz et al., 2025).

### 10.2. Anti-hypertensive and cardiometabolic relevant peptides

A fermentation-based bioprocess utilizing proteolytic *Bacillus subtilis* strains has been developed to enhance the functional properties of rice bean (*V. umbellata*) through the release of bioactive peptides. During fermentation, enzymatic hydrolysis of rice bean proteins produced short peptides, which were characterized using LC–MS/MS, revealing distinct strain-specific profiles. Functional prediction indicated that antihypertensive peptides were the most abundant category (3.90%), with hydrolysates from *B. subtilis* KN2B exhibiting the highest angiotensin-converting enzyme (ACE)-inhibitory activity (45.73%) in enzymatic assays. Molecular docking further validated peptide functionality, with nineteen peptides evaluated for ACE binding affinity. Notably, the proline-rich sequences PFPIPFPIPIPLP (common) and IPFPPIPFLPPI (unique to KN2B) displayed strong interactions within the ACE active site, supported by favorable binding energies. The prevalence of proline residues in the C-terminal tripeptide region, a recognized structural feature of effective ACE inhibitors, likely contributes to gastrointestinal stability and functional persistence. These findings, supported by *in vitro* enzymatic assays and *in silico* modeling rather than human trials, underscore the importance of microbial strain selection in shaping peptide diversity and cardiometabolic nutrition relevant functionality. Fermentation of rice bean therefore represents a sustainable strategy for generating peptides linked to cardiometabolic nutrition, with translational relevance for functional food and nutraceutical development (Padhi et al., 2022).

### 10.3. Glycemic regulation relevant phenolic acids and flavonoids

Rice bean exhibits glycemic regulation relevant potential attributable to its rich and diverse phytochemical composition. Seeds accumulate substantial levels of phenolic acids including *p*-coumaric, ferulic, and sinapic acids and flavonoids such as catechin, epicatechin, vitexin, isovitexin, and quercetin, with vitexin and isovitexin reaching 401.84 μg/g and 190.29 μg/g, respectively, in the most enriched genotypes. These compounds impart potential antioxidant capacity, with DPPH scavenging activity ranging from 39.87 to 46.40 μM TE/g, strongly correlating with total phenolic and flavonoid content based on *in vitro* assays. Antioxidant defense is critical in diabetes, mitigating hyperglycemia-induced oxidative stress that contributes to insulin resistance, β-cell dysfunction, and vascular injury. Beyond redox modulation, rice bean extracts inhibited α-glucosidase by 44.32–68.71%, with the most active genotype reaching 70% inhibition in enzyme-based assays, comparable to the pharmaceutical agent acarbose, thereby attenuating postprandial hyperglycemia. Additionally, rice bean suppressed advanced glycation end product (AGE) formation by 34.11–75.75% in BSA glucose and BSA–MGO assays, with top-performing varieties exceeding 65% inhibition. These antiglycation and enzyme inhibitory activities, supported by compositional, enzymatic, and preclinical studies rather than human trials, are mechanistically relevant to dietary glycemic regulation. Variability in α-glucosidase inhibition independent of total phenolic content further suggests contributions from specific phenolic subclasses or non-phenolic constituents, underscoring the need for targeted metabolite bioactivity mapping. Collectively, rice bean integrates antioxidant defense, digestive enzyme modulation, and antiglycation activity into a nutrition-relevant framework, positioning it as a promising functional food candidate rather than an established antidiabetic intervention (Yao et al., 2012).

### 10.4. Modulators of tissue repair relevant pathways

Rice bean is a rich source of plant proteins that, upon enzymatic hydrolysis, yield bioactive peptides with mechanistic relevance to tissue repair–associated pathways, as inferred primarily from *in vitro* studies. In recent studies, rice bean proteins digested with pepsin, trypsin, chymotrypsin, and thermolysin produced hydrolysates exhibiting significant antioxidant activity, with crude extracts showing 35.11% and hydrolysates 19.68% DPPH radical scavenging after 4 h of hydrolysis. Such antioxidant capacity is relevant to redox balance during tissue remodeling, as oxidative stress can impair fibroblast, keratinocyte, and endothelial cell function. Beyond radical scavenging, rice bean derived peptides are hypothesized based on compositional features and pre-clinical observations to influence extracellular matrix stability, collagen synthesis, inflammatory signaling, and cell migration. The enrichment of hydrophobic and aromatic amino acid residues further suggests potential gastrointestinal stability and bioactivity. Taken together, these findings indicate that rice bean derived peptides constitute a sustainable source of antioxidant bioactives with preclinical and mechanistic relevance for functional foods and nutraceuticals targeting oxidative stress associated tissue maintenance, rather than validated therapeutic wound healing agents (Grijaldo et al., 2022).

## 11. Omics-driven crop improvement and systems biology

### 11.1. Conventional and molecular breeding strategies

Domestication genetics indicate that a limited number of major-effect loci, often clustered in QTL regions such as those mapped on Linkage Group 4 (LG4), govern these traits, showing extensive collinearity with adzuki bean and thus facilitating candidate gene identification through comparative genomics within *Vigna* (Isemura et al., 2011). Broad germplasm surveys across Asia, encompassing both wild and cultivated accessions, underscore substantial variation in earliness, seed size, and local adaptation, offering immediate opportunities for direct selection, pure-line development, and pyramiding of desirable alleles (Tian et al., 2013). Pedigree-based selection following controlled hybridization remains the principal approach for combining early maturity, reduced pod shattering, and improved plant architecture, supported by precise phenotyping for flowering time, pod dehiscence, and growth habit (Isemura et al., 2010). Inter-specific hybridization with related *Vigna* (e.g., adzuki, mungbean) using embryo rescue techniques demonstrates the feasibility for introgression of useful alleles, despite variable fertility outcomes (Chen et al., 1983). Additionally, induced mutagenesis via EMS and gamma irradiation has generated agronomically relevant variants in plant type, maturity, and seed traits, with optimized protocols enhancing mutagenic efficiency for pre-breeding applications (Bhoi and Mishra, 2021).

A study addressed domestication-related traits in rice bean, developing a linkage map across 11 linkage groups and identifying clustered QTLs of large effect for seed size, pod shattering, and growth habit. Importantly, this map demonstrated strong genome collinearity with adzuki bean, facilitating candidate gene transfer between species (Isemura et al., 2010). Postharvest bruchid infestation represents a major constraint, and resistance QTLs identified in rice bean provide robust markers to track resistance alleles during backcrossing into elite genetic backgrounds (Venkataramana et al., 2016). Advances in transcriptomics have yielded gene-based SSR markers (via Illumina RNA-seq) that are now widely deployed for diversity assessments, mapping populations, and marker-assisted selection (MAS), complemented by genome-wide SSR surveys (Chen et al., 2016). Comparative genomics reviews across *Vigna* highlight conserved domestication loci (e.g., determinacy, pod shattering), providing transferable candidates for rice bean improvement (Verma et al., 2022). Strategic deployment of SSR/SNP markers flanking domestication and resistance QTLs can accelerate backcrossing while maintaining landrace-preferred traits (Venkataramana et al., 2016). With growing SNP/SSR datasets, genomic selection (GS) models for polygenic targets such as earliness, yield, and architecture are becoming feasible (Dwivedi et al., 2023). Finally, embryo rescue, bridge crosses, and induced mutagenesis (EMS, gamma irradiation) coupled with MAS or GWAS. It enable the identification and introgression of favorable alleles for growth habit, maturity, and shattering (Mishra et al., 2019).

### 11.2. CRISPR, marker-assisted selection, and metabolite-driven selection

Despite significant advances in legume functional genomics, *V. umbellata* still lacks peer-reviewed reports of successful *in-planta* CRISPR/Cas-mediated genome edits. Nonetheless, enabling resources are now available, including high-quality reference genomes, candidate domestication loci, transcriptomic datasets, and increasing legume genome-editing expertise, making translational application from close *Vigna* relatives increasingly feasible (Kaul et al., 2022; Nivya and Shah, 2023). Lessons from established CRISPR pipelines in closely related species such as mung bean, black gram, and cowpea offer validated transformation systems (Agrobacterium-mediated, hairy root, and protoplast-based assays) and multiplex editing strategies that can be directly adapted for rice bean. A notable example is the resolution of a domestication polymorphism regulating determinate versus indeterminate growth habit in rice bean landraces, linked to orthologs of *TFL1/PRR*-like regions identified through GWAS and selection scans (Guan et al., 2022; Li et al., 2018b). This represents a clear candidate for editing or allele-transfer to create semi-determinate growth, which can improve mechanized harvesting. Similarly, orthologs governing pod shattering identified in QTL and domestication studies (Isemura et al., 2010) represent critical editing targets for developing reduced-shattering ideotypes. Beyond architecture, seed quality improvement is feasible through base-editing of anti-nutritional factor pathways, such as raffinose family oligosaccharide (RFO) biosynthesis genes (*raffinose synthase*, *galactinol synthase*), as demonstrated in other legumes (Kiryushkin et al., 2022; Bridgeland et al., 2023). CRISPR–Cas systems targeting begomo viruses have demonstrated efficacy in mung bean, notably against mungbean yellow mosaic virus (MYMV), and similar vector frameworks can be rationally extended to *V. umbellata* for parallel resistance strategies (Talakayala et al., 2022). A pragmatic workflow tailored to *Vigna*’s recalcitrant tissue culture involves sgRNA design based on the rice bean reference genome, with functional validation in protoplasts or hairy-root assays (Kiryushkin et al., 2022; Bridgeland et al., 2023). Subsequent integration can leverage Agro-bacterium- or EA-mediated transformation pipelines optimized from cowpea and mung bean. Edited T_0_ plants are screened, followed by outcrossing for transgene-free lines (Che et al., 2021), with phenotypic evaluations in short-day nurseries and edit stacking via marker-assisted selection (MAS) (Sainger et al., 2015).

High-quality reference genomes and landrace resequencing further provide scans for adaptation and selection, yielding SNP panels for background recovery and fine-mapping (Kaul et al., 2022). An ideotype combining semi-determinacy, reduced shattering, photoperiod insensitivity, and bruchid resistance can be developed by integrating domestication and bruchid QTL markers with TWAS-derived SNPs (Sahu et al., 2024b), while genome-wide SNPs from landrace studies accelerate recovery in pre-breeding programs (Guan et al., 2022).

An integrated breeding framework for rice bean can strategically combine marker-assisted selection (MAS), metabolite profiling, and CRISPR editing. MAS can be deployed immediately using domestication and bruchid resistance QTLs, together with TWAS-derived markers, to assemble improved agronomic backgrounds (Jiang et al., 2023). Metabolite-driven selection provides a parallel layer, enabling enhancement of nutritional and processing traits such as increased phenolic content and reduced raffinose family oligosaccharides (RFOs) without requiring gene edits (Jiang et al., 2023). In tandem, CRISPR offers precision for traits with simple genetic control, including determinacy, pod shattering, and RFO enzyme pathways, with edits validated through protoplast or hairy-root assays before scaling into full transformation pipelines (Kiryushkin et al., 2022; Bridgeland et al., 2023).

### 11.3. Genetic potential and breeding constrains of rice bean

*V. umbellata*, a climate-resilient and nutrient-rich legume, has significant potential to contribute to food security, rural livelihoods, and sustainable agriculture, yet it remains underexploited relative to major pulses. Targeted breeding for high-yielding, early-maturing, and disease-resistant cultivars is essential to expand their cultivation and ensure adaptability across diverse agroecological zones (Guan et al., 2022). Breeding priorities for *V. umbellata* include determinacy, synchronized maturity, seed uniformity, improved palatability, and yield stability—traits essential for both farmer adoption and consumer acceptance. Recent genomic advances, including transcriptomic datasets and draft genome assemblies, offer unprecedented opportunities for molecular breeding. Strengthened global collaboration, participatory breeding, and strategic investment could reposition rice bean as a climate-resilient, nutritionally superior crop (Pattanayak et al., 2019).

### 11.4. Omics-driven discoveries: genomics, transcriptomics, metabolomics, and proteomics

Recent genomic advances unlock the genetic blueprint of *V. umbellata*, establishing critical resources for modern breeding. A high-quality reference genome, coupled with population resequencing of landraces, has identified genomic regions associated with key domestication traits such as seed size, pod dehiscence, twining habit, and flowering time. Foundational linkage maps and domestication QTLs revealed that a few major loci explain much of the variation in these traits, with strong synteny to adzuki bean, thereby facilitating candidate-gene transfer and comparative mapping. Collectively, these resources provide a scaffold for marker-assisted selection (MAS), genomic selection (GS), and CRISPR/Cas gene-editing workflows. The QTL maps and landrace resequencing yield concrete loci and functional markers for targeted trait improvement (Guan et al., 2022; Isemura et al., 2010). Complementing this, transcriptomic studies have expanded rice bean gene catalogs through de novo assemblies and RNA-seq, uncovering key transcription factor families, WRKY, MYB, and NAC that regulate stress responses and secondary metabolism. Transcriptome-derived SSRs offer additional markers for diversity analysis and mapping, while Transcriptome-Wide Association Studies (TWAS) have identified expression-linked candidate genes for flowering, maturity, and seed weight, with potential for conversion into breeder-friendly assays (Sahu et al., 2024b). Comparative transcriptomics across *Vigna* species highlights stress-responsive pathways underlying rice bean’s resilience, reinforcing its value as a “climate-smart” legume. By bridging genotype to phenotype, transcriptomics provides insight into which loci are functionally expressed under specific developmental stages or stresses, generating prioritized targets for MAS, CRISPR validation, and transgenic interventions that can accelerate the crop’s genetic improvement (Chen et al., 2016).

### 11.5. Novelty and conceptual advancement: integrating AI-driven crop design with ADMET-informed food–pharma translation

The central novelty of this review lies in its explicit conceptual integration of two traditionally disconnected research domains—AI-driven crop design and pharmaceutical ADMET (Absorption, Distribution, Metabolism, Excretion, and Toxicity) profiling—applied to Rice Bean (*V. umbellata*) within a unified translational framework. Unlike existing literature that typically addresses agronomic improvement, genomic breeding, or nutritional composition in isolation, this review advances a cross-disciplinary bridge linking plant systems intelligence with drug-likeness, bioavailability, and metabolic fate assessment, thereby extending the analytical scope of food composition research toward translational health relevance (Wang et al., 2025).

#### 11.5.1. Systems biology and AI as the enabling foundation

Systems biology provides the mechanistic backbone for this integration by bridging genotype to complex phenotype through the incorporation of multi-omics datasets, including genomics, transcriptomics, proteomics, and metabolomics, into predictive computational models. These models enable simulation of complex trait networks and identification of key regulatory nodes governing nutrient-use efficiency, stress resilience, photosynthetic performance, and plant architecture. By elucidating molecular pathway interactions underlying these traits, systems biology facilitates the rational design of “smart crops” with optimized trait combinations, accelerating targeted breeding and precision biotechnology interventions (Kumar et al., 2015).

Artificial intelligence (AI) further amplifies this framework by enabling the integration and analysis of large-scale, high-dimensional datasets, combining multi-omics, phenomic, and enviromic information to uncover hidden patterns and predictive relationships. Within a systems biology context, AI can model network behavior and predict phenotypic outcomes across genomic, transcriptomic, proteomic, and metabolomic layers. Contemporary “Breeding 4.0” paradigms leverage AI alongside high-throughput phenotyping platforms such as sensor arrays and UAV-based imaging—to accelerate trait prediction and selection. This data-driven approach empowers breeders to holistically optimize crop performance, resilience, and nutritional quality at scale (Zhang et al., 2025).

#### 11.5.2. Enviromics driven precision breeding and context specific optimization

Enviromics represents a further transformative dimension by integrating environmental variables—including soil characteristics, climate parameters, and management practices—into predictive genotype-to-phenotype models, enabling robust dissection of trait plasticity and genotype × environment (G×E) interactions (Xu et al., 2022; Resende et al., 2021). AI-driven enviromics pipelines facilitate the design of environment-specific genotypes, streamline introgression and gene-editing strategies, and reduce breeding cycle duration while conserving resources. Analogous implementations in major crops, such as CNN–RNN frameworks integrating genotype and weather data for yield prediction, demonstrate the feasibility of high-accuracy performance forecasting across agroecological contexts. The convergence of AI, big data, multi-omics, and enviromics thus aligns strongly with “Breeding 4.0” principles and provides a systems-level, precision-driven strategy for accelerating the development of resilient, high-performing rice bean cultivars (Zhang et al., 2025).

#### 11.5.3. Translational extension to ADMET informed food–pharma evaluation

Most prior studies on rice bean and related legumes have focused either on agronomic and genomic dimensions, such as yield components, flowering behavior, stress adaptation, and genetic diversity, or on nutritional and phytochemical profiling, emphasizing macronutrients, micronutrients, and antioxidant potential for functional food applications. In contrast, the present review uniquely extends AI-enabled systems biology and enviromics-informed breeding into the post-harvest and post-consumption domain by embedding in silico pharmacokinetic and ADMET evaluation of rice bean–derived bioactives. This integration enables systematic linkage of genotype-informed crop design with predicted absorption, metabolism, bioavailability, and safety profiles of food-derived metabolites, a connection largely absent from current food composition literature (M et al., 2026).

This review introduces a first-of-its-kind translational framework that bridges AI-enabled crop ideotype design with in silico pharmaco-kinetic and ADMET profiling of Rice Bean

(*V. umbellata*) bioactives, positioning the crop not only as a climate-resilient orphan legume but also as a computationally optimized source of bioavailable, drug-like phytochemicals with functional food and therapeutic relevance.

While previous studies have independently explored (i) AI and machine-learning approaches for crop trait prediction and yield optimization, (ii) genomic and transcriptomic analyses of rice bean adaptation and stress resilience, and (iii) phytochemical and nutraceutical characterization for food and health applications, no prior synthesis has systematically integrated these domains to (a) connect AI-guided crop design with predicted pharmacokinetic behavior of food-derived metabolites, or (b) map rice bean metabolite diversity directly onto ADMET-relevant chemical space for compound prioritization (Tomar, 2025).

Core conceptual innovations highlighted in this review is as follows

- AI-driven crop design is reframed beyond yield and stress tolerance to include metabolite-aware ideotype engineering, enabling selection of genotypes enriched in bioactives with favorable absorption and metabolic profiles (van Eeuwijk et al., 2019).
- ADMET profiling is extended from synthetic chemical libraries to plant-derived phytochemical systems, embedding pharmacokinetic and safety considerations directly within food composition analysis and breeding pipelines (Daina et al., 2017).

Rice bean is positioned as a dual-use computational bioresource, simultaneously supporting, climate-resilient agricultural development, and evidence-based functional food innovation and early-stage drug discovery.

By establishing a previously missing interdisciplinary interface—from crop genomics, AI-based phenotyping, and enviromics-informed breeding to phytochemical prediction and ADMET-based candidate filtering, this review delivers a systems-level framework that aligns food composition analysis with precision nutrition and translational health science. This integrative perspective broadens the functional interpretation of crop composition data and underscores the emerging role of AI in connecting sustainable agriculture with human health outcomes.

## 12. Functional food, nutraceutical, and bioactive delivery applications

Rice bean’s drug delivery potential is derived from its polysaccharides and resistant starch, while its nutraceutical promise is underpinned by phenolics and isoflavones with synergistic effects. Pre-clinical studies indicate significant bioactivities; however, critical knowledge gaps remain, including standardized extraction protocols, bioavailability assessment, and rigorous clinical validation.

### 12.1. Drug delivery applications

Legume-derived polysaccharides and proteins are increasingly explored as biodegradable carriers for oral, colon-targeted, and nanoparticle-based delivery, owing to their mucoadhesive properties and encapsulation capacity for bioactives (Salehi and Rashidinejad, 2025). Physical and chemical modifications including pregelatinization, phosphorylation, and carboxymethylation enable tailored physicochemical properties for specific dosage forms. Carboxymethylated rice bean starch (CMRBS) exhibits enhanced compressibility, tablet hardness, and elevated resistant starch content (21.65%), supporting its use in binding and controlled-release formulations. Pregelatinized starch (PGRBS) provides high free swelling capacity, suitable for immediate-release applications, while phosphorylated starch (CLRBS) offers excellent flow properties (Carr’s Index 12.16%, Hausner ratio 1.14%), facilitating uniform dosing and industrial scalability. Collectively, these functionalities, including binding, disintegration, solubility enhancement, and flow regulation, position rice bean starch as a competitive plant-based alternative to conventional excipients. Beyond pharmaceuticals, its integration into health-oriented foods and nutraceuticals underscores the valorization of this underutilized legume, promoting sustainable innovation across both industrial and nutritional sectors (Kittipongpatana et al., 2025).

### 12.2. Synergistic nutraceuticals

The rice bean is rich in phenolics such as catechins, rutin, and quercetin, alongside with isoflavones, essential amino acids, and minerals. Its polyphenols exhibit strong antioxidant activity and may synergize with other legumes or cereals in mixed diets to enhance bioactivity (Zhang et al., 2020). Furthermore, integrating rice bean proteins with probiotics or omega–3 fatty acids can enable multi-target nutraceutical formulations, providing complementary benefits for metabolic health and chronic disease management, thereby extending the functional and therapeutic potential of this underutilized legume beyond single-compound applications (Leena et al., 2020).

### 12.3. Functional food development based on composition-driven properties

Rice bean (*V. umbellata*) represents a compositionally rich legume with significant potential for functional food development due to its integrated profile of macronutrients, micronutrients, and bioactive phytochemicals. Its macronutrient composition, including moderate-to-high protein (17–25%), complex carbohydrates (~61–65%), low lipid content (~3.5–4.0%), and dietary fiber (5–7%), provides a nutritionally balanced foundation for energy-dense formulations (Katoch, 2013; Kaur et al., 2013). The protein fraction, enriched in globulins and albumins and containing ~40% essential amino acids, particularly lysine, enhances its complementarity with cereal-based diets (Katoch et al., 2023; Rodriguez and Mendoza, 1991; Yao et al., 2012). Rice bean also contains diverse phenolic compounds, including phenolic acids and flavonoids such as quercetin, rutin, catechin, and taxifolin, contributing to its antioxidant potential. A substantial proportion of bound phenolics (~71%) suggests that processing methods strongly influence its functional expression (Jiang et al., 2023). Enzymatically derived peptides exhibit in vitro bioactivities, including ACE-inhibitory and radical-scavenging effects, indicating mechanistic nutritional relevanc (Wang et al., 2014; Gonzàlez-Cruz et al., 2025). However, these findings remain limited to preclinical models. Micronutrient density, particularly iron, calcium, and phosphorus, further supports its nutritional value, although bioavailability is constrained by phytic acid and tannins. Processing techniques such as fermentation, germination, and thermal treatment improve digestibility and enhance bioactive release (Salim et al., 2023; Sarra et al., 2026). Collectively, rice bean’s functional potential is composition-driven and processing-dependent, warranting further validation through bioavailability and clinical studies (Salehi and Rashidinejad, 2025; Keskin et al., 2022).

## 13. Limitations and translational gaps in rice bean bioactivity research

Despite increasing interest in the bioactive potential of rice bean (*V. umbellata*), the current body of evidence remains largely preliminary and heterogeneous, warranting cautious interpretation (Ahmed and Jamil, 2024). Much of the literature reporting antioxidant activity is based on *in vitro* chemical assays such as DPPH, ABTS, FRAP, and ORAC. While these methods are useful for compositional screening and comparative purposes, they are not designed to predict biological activity in complex physiological systems. Consequently, antioxidant capacity values derived from these assays should be interpreted strictly as indicators of redox-reactive constituents rather than as direct evidence of health-promoting effects (Yenduri et al., 2026).

A major challenge in synthesizing existing findings is the substantial methodological variability across studies. Reported differences in antioxidant and enzyme-inhibitory activities are strongly influenced by genotype, geographical origin, agroclimatic conditions, post-harvest processing, extraction solvents, and analytical protocols (Devkota et al., 2023). The lack of standardized extraction procedures, reference standards, and harmonized reporting unit limits reproducibility and restricts meaningful quantitative comparison across datasets. As a result, reported bioactivity values may reflect analytical conditions rather than intrinsic compositional differences (Reichenpfader et al., 2025).

Evidence supporting antidiabetic potential is similarly constrained. Most studies rely on *in vitro* inhibition of carbohydrate-hydrolyzing enzymes (α-amylase and α-glucosidase) or short-term animal models of hyperglycemia. Although such approaches provide initial indications of potential glycemic modulation, they offer limited insight into long-term metabolic regulation, insulin sensitivity, or postprandial glucose dynamics in humans. Moreover, inhibitory effects are frequently observed at extract concentrations that may not be achievable through realistic dietary consumption, raising concerns regarding physiological relevance. Inconsistencies in reported outcomes may further reflect the influence of antinutritional components, food matrix effects, and differences in processing or formulation (Yao et al., 2012).

Reports of anti-inflammatory or cytoprotective effects remain sparse and are largely confined to cell-based assays or acute animal studies, often without comprehensive mechanistic validation. Importantly, human intervention data are currently lacking. The absence of controlled clinical studies represents a significant translational gap, limiting the ability to relate compositional findings to functional or health outcomes (Gonzàlez-Cruz et al., 2025).

From a food composition perspective, these limitations highlight the need to clearly distinguish between compositional bioactivity potential and demonstrated biological efficacy. Future research should emphasize standardized analytical methodologies, detailed compositional characterization, and dose-relevant assessment of bioactive constituents. Where possible, *in vitro* findings should be contextualized using physiologically relevant models and aligned with realistic dietary exposure scenarios. Such rigor is essential to ensure that conclusions regarding the functional relevance of rice bean bioactives remain evidence-based and proportionate to the strength of the available data (Table 5).

## 14. Prospective applications based on compositional and functional insights

Rice bean (*V. umbellata*) exhibits a diverse compositional profile, including starch, dietary fiber, and phenolic constituents, whose physicochemical characteristics are theoretically relevant to a range of food, nutraceutical, and pharmaceutical applications. However, it is important to emphasize that most evidence supporting such applications is inferred from compositional and functional analyses rather than from direct performance based evaluation (Katoch et al., 2023).

Rice bean starch has been characterized in terms of granule morphology, amylose–amylopectin ratio, gelatinization temperature, swelling capacity, and pasting behavior. These properties are suggestive of potential utility in structured food systems and may, by analogy with other legume starches, be theoretically applicable to excipient or carrier functions (Keskin et al., 2022). Nevertheless, rice bean specific data on critical pharmaceutical parameters such as compressibility, flowability, tablet disintegration, drug–polymer compatibility, and controlled-release behavior are currently lacking. As such, any proposed pharmaceutical application should be regarded as requiring further validation.

Similarly, discussion of rice bean-derived polysaccharides or modified starches in drug delivery contexts is largely extrapolated from broader literature on plant-based biopolymers. While compositional features such as hydrophilicity, molecular weight distribution, and gel-forming ability are *suggestive* of carrier potential, these characteristics alone are insufficient to establish suitability for drug delivery systems. Rigorous assessment through *in vitro* release studies, stability testing, biocompatibility evaluation, and comparative benchmarking against established excipients would be necessary to substantiate such claims (Salehi and Rashidinejad, 2025).

From a nutraceutical perspective, rice bean phytochemicals and complex carbohydrates may be theoretically relevant for incorporation into functional formulations. However, current studies primarily report compositional abundance or *in vitro* bioactivity without addressing formulation stability, processing losses, matrix interactions, or bioavailability enhancement. Consequently, nutraceutical applicability should be interpreted as prospective rather than demonstrated.

Overall, while the compositional attributes of rice bean provide a rational basis for exploring diverse applications, existing evidence remains insufficient to support definitive claims beyond food composition and basic functionality.

## 15. Conclusions and future perspectives

Looking ahead, the strategic advancement of rice bean requires a decisive shift from descriptive characterization toward integrative, systems-level innovation. Future research must prioritize multi-omics integration linking genomics, transcriptomics, metabolomics, and proteomics with high-resolution phenoltyping to unravel genotype–phenotype environment interactions that govern nutritional quality, stress resilience, and agronomic performance. Embedding these datasets within systems biology and AI-assisted predictive frameworks will accelerate ideotype design tailored to marginal agroecosystems and nutrition-sensitive agriculture. Equally critical is the translation of omics discoveries into farmer-ready outcomes through participatory breeding, decentralized seed systems, and value-chain integration. Aligning scientific advances with policy incentives, climate-resilient farming models, and functional food innovation will ultimately determine whether rice bean transitions from an orphan legume to a cornerstone crop in future-ready food systems. While this narrative synthesis highlights substantial compositional and omics-driven potential, translation to clinical and dietary recommendations will require well-designed human studies. The roadmap forward is thus not incremental, but transformative anchored in multi-omics convergence, socioecological relevance, and translational impact.

## Figures and Tables

**Table 1:** Major nutraceutical-relevant compounds reported in rice bean (*Vigna umbellata*).

Compound class	Representative constituents	Reported range / trend*	Primary compositional relevance	Key analytical notes / limitations
Phenolic acids	Gallic acid, ferulic acid, p-coumaric acid	Variable; genotype- and extraction-dependent	Contribute to total phenolic content and redox reactivity	Quantification highly dependent on solvent system and assay method
Flavonoids	Catechin, quercetin derivatives, kaempferol derivatives	Generally higher in seed coat fractions	Influence antioxidant assay outcomes	Often reported as total flavonoid equivalents; limited compound-specific profiling
Tannins	Condensed tannins (proanthocyanidins)	Higher in pigmented varieties	Affect antioxidant values and protein interactions	Dual role as antioxidant and anti-nutritional factor
Dietary fiber	Insoluble and soluble fiber fractions	Moderate to high	Relevant to carbohydrate quality and gut-related functionality	Limited differentiation between fermentable and non-fermentable fractions
Resistant starch	Native and retrograded starch fractions	Processing-dependent	Influences glycemic response potential	Few standardized methods used across studies
Phytates	Phytic acid (IP6)	Relatively high, typical of legumes	Major determinant of mineral bioavailability	Often not reported alongside mineral data
Oligosaccharides	Raffinose, stachyose	Present at moderate levels	Relevant to fermentability and digestive tolerance	Limited profiling of individual saccharides

* Reported values vary widely across studies due to differences in genotype, environment, processing, and analytical methodology.

**Table 2:** Table illustrating micronutrient quantities in rice bean (*Vigna umbellata*) showing genotype dependent variability in nutrient contents per 100 g and also their functions.

Micronutrient	Rice Bean (*V. umbellata*)	Mung Bean (*V. radiata*)	Black Gram (*V. mungo*)	Functions
Calcium (Ca)	~200 mg (68–230 mg range)	Moderate (Not specified)	~138 mg	Bone health, muscle contraction
Phosphorus (P)	~390 mg (209–370 mg range)	Moderate	-	Energy metabolism, bone structure
Iron (Fe)	~10.9 mg	-	~7.6 mg	Hemoglobin formation, oxygen transport
Potassium (K)	-	-	~983 mg	Nerve signaling, fluid balance
Magnesium (Mg)	-	Present	-	Enzyme function, muscle health
Thiamin (Vitamin B_1_)	~0.49 mg (0.5–1.09 mg range)	Present	-	Carbohydrate metabolism, nerve health
Riboflavin (Vitamin B_2_)	~0.21 mg (0.18–0.5 mg range)	Present	Cultivar-specific high	Energy production, cellular metabolism
Niacin (Vitamin B_3_)	~2.4 mg (2.0–3.6 mg range)	~3.87 mg	-	Metabolism, skin and nerve function
Ascorbic Acid (Vitamin C)	Up to 23.46 mg	Present	-	Antioxidant, immune support
Folate (Vitamin B_9_)	-	-	~628 μg	DNA synthesis, cell division
Pantothenic Acid (B_5_)	-	Present	-	Energy metabolism, hormone synthesis
Tocopherols (Vitamin E)	-	Present	-	Antioxidant, cell protection
Dietary Fiber	5–7% of seed weight	Moderate	Elevated	Gut health, bowel function

**Table 3:** Illustrates the phytochemicals by comparing profiles of Rice bean (*Vigna umbellata*) and its genotypes-Mung bean (V. radiata), Black gram (V. mungo), and Soybean (Glycine max)-representing variety in phenolic contents and flavonoids. It also shows key bioactive compounds consisting of antioxidant, anti-diabetic and other properties.

Category	Compounds	Rice bean (*V. umbelleta*)	Mung bean (*V. radiate*)	Black gram (*V. mungo*)	Soyabean (Glycine max)	Functions
Total Phenolic Content (TPC)	-	2–5 mg GAE/g; Bound phenolics ~71% (14.18 mg GAE/g)	Moderate; rich in protocatechuic & chlorogenic acids	Moderate to high: contains vanillic & protocatechuic acids	Moderate	Antioxidant, anti-inflammatory
Phenolic Acids	Gallic, Caffeic, Ferulic, p-Coumaric	Present in abundance	Protocatechuic, Chlorogenic	Gallic, Caffeic, Ferulic, Vanillic, Protocatechuic, p-Coumaric	Isoflavones dominate instead	Antioxidant & metabolic regulation
Bound Phenolics	Gallic acid, Protocatechuic acid, p-Hydroxybenzoic acid, Catechin, Phloroglucinol	Dominant fraction (~71%)	Moderate	-	-	Strong antioxidant potential
Free Phenolics	Isoquercitrin, Procyanidin B_1_, Rutin, Taxifolin, Catechin	Abundant	Moderate	-	-	Free radical scavenging, metabolic protection
Flavonoids	Quercetin, Rutin, Isoquercitrin, Kaempferol–3-O-rutinoside, Taxifolin, Catechin, Procyanidin B_1_	Abundant & diverse	C-Glycosyl flavones (Orientin, Isoorientin, Vitexin, Isovitexin)	Quercetin, Kaempferol, Rutin, Apigenin, Luteolin, Vitexin	Isoflavones (Genistein, Daidzein, Glycitein 5–10%)	Antioxidant, anti-inflammatory, cardioprotective
Dominant Isoflavones	-	Absent	Absent	Absent	Genistein, Daidzein, Glycitein	Phytoestrogenic activity, hormonal regulation
Tannins	-	~1.4–1.6% dry weight	Moderate	Moderate	Moderate	Antinutritional, antioxidant role
Trypsin Inhibitors	-	Present	Present	Present	Present	Antinutritional, protein digestion interference
Phytic Acid & Saponins	-	Present	Present	Present	Present	Mineral-binding, cholesterol-lowering effects
Protein-Derived Peptides	Antifungal peptides (active vs. *B. cinerea, F. oxysporum, R. solani*)	Present	-	-	-	Antifungal, mitogenic, anti-HIV–1 RT activity
Anti-oxidant Activity (in vitro)	Phenolic extracts & hydrolysates	Strong (via free & bound phenolics)	Moderate	Strong	Moderate	Scavenges reactive oxygen species
Anti-proliferative Activity	Against MDA (breast) & SiHa (cervical) cancer cell lines (IC_50_ ~1000 μg/mL)	Reported	-	-	-	Cancer-preventive potential
Enzymatic Inhibition	α-Glucosidase, Dipeptidyl Peptidase	Inhibitory	-	-	-	Anti-diabetic potential
Hepato-protective Effect	Against acetaminophen toxicity	Present	-	-	-	Liver protection

**Table 4:** Table showing phytochemicals in certain regions of Asia and their functions and traditional uses in those regions.

Region	Phytochemicals	Functions	Cultural uses
China	Quercetin, Catechin, Ferulic Acid	Antioxidant, Antidiabetic	Used in local functional food studies
India	Vitexin, Isovitexin, p-Coumaric acid	Immunomodulatory, Digestive	Local landraces, high-protein pulses
Nepal	Tannins, Ferulic, Sinapic acids	Antioxidant, Glycemic control	Ingredient in *Kwati*, fasting ritual food
Thailand/Laos	Anthocyanins, Tannins	Antioxidant, Anticancer	Pigmented seeds, traditional cuisine
Japan(imported)	Isoflavones, Resistant Starch	Hormonal balance, Cardiovascular health	Imported, used in low-GI diets

**Table 5:** Summary of evidence strength and methodological constraints in Rice Bean bioactivity studies.

Model system	Target activity	Representative approaches	Key limitations
*In vitro* (chemical assays)	Antioxidant	DPPH, ABTS, FRAP, ORAC	Reflect chemical reactivity rather than biological function; highly sensitive to extraction and assay conditions
*In vitro* (enzyme-based)	Antidiabetic	α-Amylase, α-glucosidase inhibition	Limited relevance to postprandial glucose regulation; often tested at non-physiological concentrations
Cell-based models	Anti-inflammatory, antioxidant	ROS modulation, cytokine assays	Limited mechanistic depth; lack of dietary exposure context
Animal models (rodent)	Antidiabetic, antioxidant, hepatoprotective	Chemically induced diabetes, oxidative stress models	Short study duration; species-dependent responses; limited translation to humans
Human studies	---	---	Currently lacking; no direct evidence linking composition to health outcomes

## Data Availability

No data was used for the research described in the article.
